# A Unified Theory of Human Judgements and Decision-Making under Uncertainty

**DOI:** 10.3390/e22070738

**Published:** 2020-07-03

**Authors:** Raffaele Pisano, Sandro Sozzo

**Affiliations:** 1 Institute of Electronics, Microelectronics and Nanotechnology (IEMN), Lille University, Avenue Poincaré CS 60069, CEDEX, 59652 Villeneuve d’Ascq, France; raffaele.pisano@univ-lille.fr; 2 School of Business and Centre for Quantum Social and Cognitive Science (IQSCS), University of Leicester, University Road, Leicester LE1 7RH, UK

**Keywords:** quantum structures, concept theory, decision theory, cognitive fallacies, disjunction effect, Ellsberg paradox, quantum modelling, historical perspectives

## Abstract

Growing empirical evidence reveals that traditional set-theoretic structures cannot in general be applied to cognitive phenomena. This has raised several problems, as illustrated, for example, by probability judgement errors and decision-making (DM) errors. We propose here a unified theoretical perspective which applies the mathematical formalism of quantum theory in Hilbert space to cognitive domains. In this perspective, judgements and decisions are described as intrinsically non-deterministic processes which involve a contextual interaction between a conceptual entity and the cognitive context surrounding it. When a given phenomenon is considered, the quantum-theoretic framework identifies entities, states, contexts, properties and outcome statistics, and applies the mathematical formalism of quantum theory to model the considered phenomenon. We explain how the quantum-theoretic framework works in a variety of judgement and decision situations where systematic and significant deviations from classicality occur.

## 1. Introduction

Set-theoretic algebraic structures, like Boolean algebras, are the building blocks of classical (Boolean) logic and classical (Kolmogorovian) probability. It is, for example, well known that the elementary connectives of conjunction, disjunction and negation in Boolean logic are represented by the set-theoretic operations of intersection ∩, union ∪ and complementation ∖, respectively. Similarly, conjunctions and disjunctions of two events are represented by the same set-theoretic operations in Kolmogorovian probability theory [1,2,3].

Set-theoretic structures were originally used in classical physics and later applied to a variety of disciplines, from psychology to economics, finance, computer science and statistics. This is why one usually refers to them as “classical structures”. In particular, Kolmogorovian probability models have characterized the experimental revolution which occurred in cognitive psychology in the 1970s [4]. In this regard, it is worth to briefly summarize the fundamental definitions and results of Kolmogorovian probability theory that are needed for the scopes of the present paper.

Let S≠∅, let P(S) be the power set of S, and let A⊆P(S). We say that A is a “σ-algebra” if it satisfies the following properties:

(I) S∈A;
(II) for every E∈A, $E^{\prime }=S\setminus E\in A$;
(III) for every countable family ${\left\{E_{i}\in A\right\}}_{i\in N}$, $\cup_{i\in N}E_{i}\in A$.

It straightly follows from the definition that, in a σ-algebra A, for every countable family ${\left\{E_{i}\in A\right\}}_{i\in N}$, $\cap_{i\in N}E_{i}\in A$. A σ-algebra A is in particular distributive, that is, for every E,F,G∈A, E∩(F∪G)=(E∩F)∪(E∩G) and E∪(F∩G)=(E∪F)∩(E∪G). It then follows from distributivity that, for every E,F∈A, $E=\left(E\cap F\right)\cup \left(E\cap F^{\prime }\right)$.

Let now S≠∅, let A⊆P(S) be a σ-algebra, and let p:E∈A↦p(E)∈[0,1] be an application over A. We say that *p* is a “normalized probability measure” if it satisfies the following conditions:

(1) p(S)=1 (normalization);
(2) for every countable family ${\left\{E_{i}\in A\right\}}_{i\in N}$ of pairwise disjoint sets, that is, $E_{i}\cap E_{j}=\emptyset ,\;i\neq j$, $p\left(\cup_{i\in N}E_{i}\right)=\sum_{i\in N}p\left(E_{i}\right)$ (additivity).

The sets in A correspond to “events” of the real world. Conditions (1) and (2) were introduced by Kolmogorov, hence, the normalized probability measure *p* is called “Kolmogorovian probability” [1].

Then, let p:E∈A↦p(E)∈[0,1] be a Kolmogorovian probability, and let E,F∈A, with p(E),p(F)≠0. We define the “conditional probability” p(E|F) of *E* given *F* as

(1) $$p\left(E|F\right)=\frac{p(E\cap F)}{p(F)}.$$

The conditional probability p(F|E) of *F* given *E* is defined in an analogous way, and the “Bayes formula”

(2) p(F)p(E|F)=p(E∩F)=p(F∩E)=p(E)p(F|E)

holds. The following laws of Kolmogorovian probability can be finally derived. For every E,F∈A,

(3) $$p\left(E\right)=p\left(F\right)p\left(E|F\right)+p\left(F^{\prime }\right)p\left(E|F^{\prime }\right),$$

which is known as the “law of total probability”, and

(4) p(E∩F)≤p(E),p(F)≤p(E∪F),

which is known as the “law of monotonicity”.

Growing empirical evidence in cognitive psychology reveals that the classical structures above, when applied to complex processes, like judgement and decision-making (DM), lead to predictions that do not agree with empirical data [4,5]. Deviations from classicality in these domains can be roughly divided into two main groups, as follows [6].

(i) Probability judgement errors. Given two events *E* and *F*, people judge the conjunction event ‘EandF’ (disjunction event ‘EorF’) as more (less) probable than the event *E* or/and *F* separately. This entails a “violation” of the of the law of monotonicity (cfr., Equation (4)).
(ii) DM errors. Given two actions *f* and *g*, people prefer *f* over *g* if they know that an event *E* occurs, and also if they know that *E* does not occur, but they prefer *g* over *f* if they do not know whether *E* occurs or not. This entails a “violation” of the law of total probability (cfr., Equation (3)).

Over-/under-extension effects in membership judgements on conceptual combinations [7,8] and conjunctive/disjunctive fallacies [9,10] are examples of type-(i) errors. The disjunction effect [11] and empirical violations of expected utility theory (EUT) [12] are instead examples of type-(ii) errors.

The existence of situations of type-(i) and type-(ii) entails, in particular, that:

- the most natural set-based models of cognition cannot in general be used to represent human judgements and decisions;
- the usual interpretation of human behaviour in terms of classical (Boolean) logic and classical (Kolmogorovian) probability theory is problematical when human judgements and decisions are at stake.

The famous “Kahneman-Tversky research programme” considers the above empirical deviations from classicality as genuine fallacies of human reasoning and provides a theoretical framework for them which assumes individual heuristics and judgement biases [5,13], in substantial agreement with Simon’s theory of bounded rationality [14]. This explains why terms like error, effect, fallacy, paradox, contradiction, and so forth, are typically used in these cases. A major limitation of the Kahneman-Tversky research programme is that it works at an intuitive level and has manifold applications, but it does not provide a general theory of human judgements and decisions. This has led various scholars to explore alternative mathematical structures which are more appropriate than classical structures to model these phenomena.

On the other side, quantum theory is certainly one of the most successful theoretical achievement that humans have ever produced. Originally elaborated to cope with specific phenomena where empirical results did not agree with the predictions of classical physics, for example, black-body radiation, photoelectric effect, atomic modelling, and so forth, quantum theory is nowadays applied to any conceivable scale, from the micro-world to living matter.

We add that classical physics had to face at the beginning of the 1900s problems of incompatibility between classical structures and their underlying logic and empirical results that are similar to the problems discussed here. For example, from the beginning of the 1900s, the problem of black-body radiation represented the critical root of quantum theory in a period in which already the space in physics had started to be conceived differently from the Euclidean one. Thus, the logical structure of the forthcoming theories changed as well. Regarding the black-body radiation, the main epistemological and foundational problem concerned the way in which the intensity of electromagnetic radiation emitted by a black body (i.e., a perfect absorber, also known as a “cavity radiator”) depended on the frequency of the radiation (i.e., the colour of light) and the temperature of the body. In other words, a mathematical function was looked for which was able to work with both energy and frequencies. This situation was logically the opposite that scientists had faced in the 1800s with the previous theory of light. Other theories such as thermodynamics, analytical theory of heat and electromagnetism were involved in the debate (see, e.g., References [15,16,17,18,19,20]).

In its modern versions, quantum theory is formulated in a mathematical language that uses Hilbert spaces, unit vectors, self-adjoint operators and unitary dynamics, which give rise to specifically non-classical algebraic and probabilistic structures, like non-Boolean lattices, non-Kolmogorovian probabilities, non-commutative algebras, and so forth. In addition, quantum effects, like contextuality, emergence, entanglement, interference, nonlocality, superposition, and indistinguishability, do not admit a classical counterpart (see, e.g., Reference [21]).

Because of their generality and flexibility, quantum structures in Hilbert space may provide the mathematical tools to deal with the classically problematical phenomenology above and, indeed, quantum mathematical structures have systematically and successfully been applied to a variety of complex systems in psychology, social science, biology and computer science (see, e.g., References [4,22,23,24,25,26,27,28,29] and references therein).

We review and deepen in the present paper the contribution to the “quantum cognition research programme” that we have provided in a decade of collaboration between the Brussels and Leicester research teams (see, e.g., References [30,31,32,33,34,35] and references therein). More precisely, we put forward a unitary theoretical framework in which both judgements and decisions are described as intrinsically non-deterministic context-dependent processes and provide the foundations for applying quantum theory in Hilbert space as a general theory for these processes. The ensuing models, originally designed to represent specific phenomena, then find their foundation and justification in the general quantum-theoretic framework presented here. The foundation of a quantum modelling can then be split into three main steps, as follows.

The first step consists in the recognition that, in any judgement/decision process, exactly as in a quantum measurement process, a contextual influence (of a cognitive, rather than physical, type) occurs where one outcome is actualized from a set of possible outcomes. Like in quantum physics, the (measurement) context does not reveal pre-existing properties of the entity but, it rather actualizes properties that were only potential in the initial state in which the entity had been prepared—unless the initial state was chosen to be an eigenstate of the measurement in object.

The second step consists in the development of a “State-Context-Property (SCoP) formalism”, originally worked out to provide a realistic and operational foundation of quantum physics [36,37]. In the SCoP formalism, tests involving judgements and decisions are considered as complex phenomena in which:

(a) the conceptual entity under study is prepared by the test in a defined state and each participant is confronted with this uniquely prepared state;
(b) an interaction occurs on a cognitive level between the conceptual entity and the participant, which acts as a (measurement) context for the entity;
(c) the interaction is in general non-deterministic and the state of the conceptual entity is transformed into a new state;
(d) the state change makes actual (potential) some properties of the conceptual entity which were potential (actual) in the initial state;
(e) when the responses of all participants are collected, a statistics of outcomes is obtained.

The third step consists in the development of a unified quantum representation. Indeed, whenever a specific cognitive phenomenon is studied and one identifies points (a)–(e) in it, namely, the conceptual entity, its states, properties, the relevant contexts and the statistics of measurement outcomes, then the notions in (a)–(e) are represented in the quantum-theoretic framework in exactly the same way as entities, states, context, properties, probabilities and dynamics are represented in the Hilbert space formalism of quantum theory. Proceeding in this way, one can in principle apply the quantum-theoretic framework to any judgement and decision.

The theoretical framework presented here is alternative to the Kahneman-Tversky research programme, because it considers the empirical deviations from classicality in cognition as natural expressions of genuine quantum structures in human reasoning, which overlap with classical reasoning and reveal a separate layer of reasoning, namely, “emergent”, or “conceptual”, reasoning [34].

For the sake of completeness, we summarize the content of this paper in the following.

We respectively review in Section 2 and Section 3 the type-(i) errors that occur in conceptual conjunctions/disjunctions and conjunctive/disjunctive fallacies. Then, we respectively review in Section 4 and Section 5 the type-(ii) errors that occur in the disjunction effect and Ellsberg-type violations of EUT. We also sketch limitations of traditional modelling techniques and explanations for both types of cognitive fallacies. Next, we present in Section 6 the SCoP formalism for conceptual entities, applying the formalism to human probability judgements in Section 6.1 and to human decisions in in Section 6.2. In these sections, we also show how a representation in Hilbert space can be constructed. This quantum-theoretic framework enables successful modelling of membership judgements in conceptual combinations, which is shown is Section 7, the conjunction and disjunction fallacy, which is shown in Section 7.1, the disjunction effect, which is shown in Section 7.2, and Ellsberg-type paradoxes, which is shown in Section 8. Finally, in Section 9, we offer some concluding remarks.

## 2. Probability Judgement Errors: Over- and Under-Extension Effects

The issue of what a concept is and how concepts combine has intrigued and fascinated epistemologists and cognitive scientists for a long time. Leaving aside the dispute between the former and the latter on the ontology of a concept, one however recognises at once the transition needed when one moves from a very abstract concept, as *Thing*, to more concrete natural concepts, as *Fruit*, …, *Apple*, …, to finally come to an object, as *This Apple*.

Concepts and categorization are the areas in cognition that deal with the ancient philosophical “problem of universals”, that is, whether unique particular objects or events can be treated “equivalently” as members of a class. According to the “classical view of concepts”, which can be traced back to Aristotelian school and has been dominant until the 1950s, all instances of a concept share a common set of necessary and sufficient defining properties. It is however well known that this classical view contrasts with some empirical results related to the way in which people concretely judge and combine concepts. In particular, the following aspects have been identified in empirical studies on categorization (see, e.g., Reference [38]).

(i) Concepts are “vague,” “fuzzy,” or “graded,” notions. For example, people judge an item like *Robin* as more typical than *Stork* when typicality is defined with respect to the concept *Bird*.
(ii) The meaning of a concept depends on the “context” in which the concept is used. For example, an item like *Snake*, or *Spider* would score a low typicality with respect to the concept *Pet*. But, if typicality is defined with respect to the concept *Weird Pet*, or *Pet of a Weird Person*, then *Snake*, or *Spider*, would score a high typicality.

To account for these features of “vagueness,” “graded typicality,” “context dependence,” several alternative theories of concepts have been proposed since the 1970s. Limiting ourselves to the most celebrated ones, we recall the following theories.

- “Prototype theory.” A given concept is associated with a “prototype,” which has a defined set of characteristic, not defining, features. Similarity with the prototype establishes the membership of an item with respect to the concept [39,40].
- “Exemplar theory.” A given concept is defined by a salient set of instances of it stored in memory [41,42].
- “Theory theory.” A concept is similar to a “mini-theory,” and concepts stand in relation to one other in the same way as the terms of a scientific theory [43,44].

Despite mutual differences, traditional theories of concepts share the view that concepts have the form of “fixed mental representations,” whereas context only plays a secondary role in all of them. On the other side, a “combination problem” raises in all traditional concept theories of concepts, namely, how the combination, for example, conjunction, disjunction, negated conjunction, of two given concepts can be consistently represented in terms of the representation of the individual concepts. The combination problem becomes even harder when the combination of more than two concepts is investigated.

At first glance, one may be tempted to believe that concepts combine by means of set-theoretic rules that are similar to those used to combine propositions in classical (Boolean) logic. In order to incorporate conceptual vagueness into such a set-theoretic perspective, Zadeh proposed to represent concepts by using “fuzzy set logic” [45]. Given a concept *A* and an item *X*, a “graded membership” $\mu_{X}\left(A\right)\in \left[0,1\right]$ exists such that, for any two concepts *A* and *B*, the conceptual conjunction ‘AandB’ satisfies the “minimum rule of fuzzy set conjunction”

(5) $$\mu \left(A\;\mathrm{and}\;B\right)=\min\left[\mu (A),\mu (B)\right],$$

and the conceptual disjunction ‘AorB’ satisfies the “maximum rule of fuzzy set disjunction”

(6) $$\mu \left(A\;\mathrm{or}\;B\right)=\max\left[\mu (A),\mu (B)\right].$$

However, a wide range of empirical findings reveal that classical set-theoretic structures and, in particular, this fuzzy set representation, are not able to model even simple combinations of two concepts.

(1) “Pet-Fish problem”, or “Guppy effect”. People judge an item like *Guppy* to be a very typical example of the conjunction *Pet-Fish*, without judging *Guppy* to be a typical example of either *Pet* or *Fish* [46].
(2) “Overextension effects in the conjunction”. For several items, people judge the membership weight of the item with respect to the conjunction to be higher than the membership weight of the item with respect to one or both the component concepts [7].
(3) “Under-extension effects”. For several items, people judge the membership weight of the item with respect to the disjunction to be lower than the membership weight of the item with respect to one or both the component concepts [8].
(4) “Borderline contradictions”. A significant number of people judge the proposition “John is tall and John is not tall” to be true, in particular, for some borderline cases of “John” [47].

In the late 1980s, James Hampton performed a series of cognitive tests on the conjunction and the disjunction of two concepts [7,8]. Let us illustrate an example of these tests in the following.

Let us consider, for example, the concepts *Fruits*, *Vegetables*, *Fruits and Vegetables*, *Fruits or Vegetables*. Let us choose a set number of items, e.g, *Apple*, *Tomato*, *Broccoli*, *Olive*, *Coconut*, *Almond*, *Raisin*, *Acorn*, *Tomato*, *Mushrooms*, and so forth. A sample of participants are asked to judge membership of a given item with respect to *Fruits*, *Vegetables* and, for example, their disjunction *Fruits or Vegetables*. A 7-point Likert scale {−3,−2,−1,0,+1,+2,+3} is used as a judgement of membership, where the positive values +1, +2 and +3 indicate that the participant considers the item to be a member of the concept, and the degree of membership increases with the value, thus the value +3 indicates that the participant considers the item to be a “strong member” of the concept. Analogously, negative numbers indicate non-membership, again in increasing order, thus the value −3 indicates that the participant considers the item to be a “strong non-member” of the concept. The value 0 indicates that the participant is unresolved about membership or non-membership of the item. The relative frequencies of positive responses are considered, in the large number limit, as probabilities of membership and are called “membership weights.”

Hampton collected membership weights of several items with respect to different pairs of natural concepts and their conjunction or disjunction, finding significant deviations from classical set-theoretic structures and introducing a specific terminology to classify such deviations.

Let *X* be an item and let μ(A), μ(B), μ(AandB) and μ(AorB) be the membership weights of *X* with respect to the concepts *A*, *B*, their conjunction ‘AandB’ and their disjunction ‘AorB’, respectively. We say that *X* is:

- “overextended with respect to the conjunction” if μ(AandB)>μ(A) or μ(AandB)>μ(B);
- “underextended with respect to the disjunction” if μ(AorB)<μ(A) or μ(AorB)<μ(B);
- “double overextended with respect to the conjunction” if μ(AandB)>μ(A) and μ(AandB)>μ(B);
- “double underextended with respect to the disjunction” if μ(AorB)<μ(A) and μ(AorB)<μ(B).

Overextension and double overextension express a violation of the law of monotonicity of Kolmogorovian probability for the conjunction of two events. Let us consider, for example, the item *Razor*. Hampton measured membership of *Razor* with respect to *Weapons*, *Tools* and their conjunction *Weapons and Tools*, finding μ(A)=0.63, μ(B)=0.78 and μ(AandB)=0.83, respectively. Hence, *Razor* is double overextended with respect to the conjunction *Weapons and Tools* [7].

However, the deviation from classical structures in conceptual conjunctions is even deeper. To illustrate this aspect, let us introduce the following definition.

We say that the membership weights μ(A), μ(B) and μ(AandB) of the item *X* with respect to the concepts *A*, *B* and their conjunction ‘AandB’, respectively, correspond to “classical data for the conjunction” if a σ-algebra A, a Kolmogorovian probability measure p:A⟶[0,1], and events $E_{A},E_{B}\in A$ exist such that:

(7) $$\begin{array}{ccc} \mu (A) & = & p(E_{A}), \end{array}$$

(8) $$\begin{array}{ccc} \mu (B) & = & p(E_{B}), \end{array}$$

(9) $$\begin{array}{ccc} \mu (A\;\mathrm{and}\;B) & = & p(E_{A}\cap E_{B}). \end{array}$$

One can then prove that μ(A), μ(B) and μ(AandB) are classical data for the conjunction if and only if the following inequalities are simultaneously satisfied:

(10) $$\begin{array}{ccc} \mu \left(A\;\mathrm{and}\;B\right)-\min\left[\mu (A),\mu (B)\right] & \leq & 0, \end{array}$$

(11) $$\begin{array}{ccc} \mu (A)+\mu (B)-\mu (A\;\mathrm{and}\;B) & \leq & 1. \end{array}$$

Inequality (10) expresses the law of monotonicity for the conjunction, while inequality (11) is called the “Kolmogorovian conjunction factor” [22].

Similarly, underextension and double underextension express a violation of the law of monotonicity of Kolmogorovian probability for the disjunction of two events. Let us consider, for example, the item *Razor*. Hampton measured membership of *Curry* with respect to *Spices*, *Herbs* and their disjunction *Spices or Herbs*, finding μ(A)=0.90, μ(B)=0.40 and μ(AorB)=0.75, respectively. Hence, *Curry* is underextended with respect to the conjunction *Spices or Herbs* [8].

However, other non-classical effects are at play in the disjunction case too. Indeed, we say that the membership weights μ(A), μ(B) and μ(AorB) of the item *X* with respect to the concepts *A*, *B* and their disjunction ‘AorB’, respectively, correspond to “classical data for the disjunction” if a σ-algebra A, a Kolmogorovian probability measure p:A⟶[0,1], and events $E_{A},E_{B}\in A$ exist such that:

(12) $$\begin{array}{ccc} \mu (A) & = & p(E_{A}), \end{array}$$

(13) $$\begin{array}{ccc} \mu (B) & = & p(E_{B}), \end{array}$$

(14) $$\begin{array}{ccc} \mu (A\;\mathrm{or}\;B) & = & p(E_{A}\cup E_{B}). \end{array}$$

One can then prove that μ(A), μ(B) and μ(AorB) are classical data for the disjunction if and only if the following inequalities are simultaneously satisfied:

(15) $$\begin{array}{ccc} \mu \left(A\;\mathrm{or}\;B\right)-\max\left[\mu (A),\mu (B)\right] & \geq & 0, \end{array}$$

(16) $$\begin{array}{ccc} \mu (A)+\mu (B)-\mu (A\;\mathrm{or}\;B) & \geq & 0. \end{array}$$

Inequality (15) expresses the law of monotonicity for the conjunction, while inequality (16) is called the “Kolmogorovian conjunction factor” [22]. Let us consider, for example, the item *Olive*. Hampton measured membership of *Olive* with respect to *Fruits*, *Vegetables* and their disjunction *Fruits or Vegetables*, finding μ(A)=0.50, μ(B)=0.10 and μ(AorB)=0.80, respectively. In this case, inequality (15) is satisfied, whereas inequality (16) is violated. Hence, the membership weights *Olive* correspond to non-classical data.

As mentioned in Section 1, over- and under-extension effects in membership judgements are examples of type-(i) errors. Additional examples of these non-classical effects will be considered in the next section.

## 3. Probability Judgement Errors: Conjunctive and Disjunctive Fallacies

The identification of fallacies in cognition and the role played by psychological insights into social science marked the beginning of new disciplines, for example, behavioural economics, and allowed Daniel Kahneman to receive the Nobel Prize in Economic Science in 2002.

One of the key results in his research, obtained by Kahneman in 1983, in collaboration with Amos Tversky, was the discovery of the “conjunction fallacy” [9]. A sample of participants underwent a test in which they were presented the following story about an hypothetical woman named “Linda”.

“Linda is 31 years old, single, outspoken and very bright. She majored in philosophy. As a student, she was deeply concerned with issues of discrimination and social justice, and also participated in anti-nuclear demonstrations.”

The test required participants to indicate which of the following events they judged as more likely.

(i) Linda is a bank teller.
(ii) Linda is a bank teller and is active in the feminist movement.

The test revealed that overall 85% of the participants judged event (ii) as more probable than event (i). This empirical pattern violates the law of monotonicity of Kolmogorovian probability (see Equation (4), Section 1). Indeed, if we denote by $p(E_{A})$ the probability that the event “Linda is a bank teller” occurs and by $p(E_{B})$ the probability that the event “Linda is active in the feminist movement” occurs, then the conjunction event “Linda is a bank teller and is active in the feminist movement” is associated with the probability $p(E_{A}\cap E_{B})$ in a Kolmogorovian probability framework, and the monotonicity law requires in particular that $p\left(E_{A}\cap E_{B}\right)\leq p\left(E_{A}\right)$, in contrast to the empirical findings in Reference [9].

The test was performed in two distinct ways.

- “Joint test”. The same group of participants were asked to judge the likelihood of both the individual event and the conjunction event.
- “Separate test”. One group of participants were asked to judge the likelihood of the individual event and another group of participants were asked to judge the likelihood of the conjunction event.

A fallacy was identified in both types of tests. A large empirical literature exists in cognitive psychology which confirms the existence of a conjunction fallacy in the “Linda story problem”. However, this is not the only probability judgement error that has been identified.

A more convincing test was performed by Morier and Borgida in 1984 [10]. People participating in the test were asked to rank the likelihood of the following events:

(i) Linda is a feminist;
(ii) Linda is a bank teller;
(iii) Linda is a feminist and a bank teller;
(iv) Linda is a feminist or a bank teller.

Let us denote by μ(A), μ(B), μ(AandB), and μ(AorB) the judgement probabilities in points (i), (ii), (iii), and (iv), respectively. Then, Morier and Borgida found that the mean probability judgements are ordered as follows:

(17) μ(A)=0.83>μ(AorB)=0.60>μ(AandB)=0.36>μ(B)=0.26.

Thus, the test revealed the simultaneous presence of a conjunction fallacy and also a “disjunction fallacy”.

As mentioned in Section 1, Kahneman and Tversky developed a research programme, assuming heuristics and biases, to account in a descriptive way how people make judgements and take decisions in the presence of uncertainty [5,9]. The main steps of it are summarized in the following.

(1) Human judgement and DM rest on a preliminary subjective assessment of the probabilities of uncertain events.
(2) In these processes, people rely on a limited number of heuristics principles, for example, representativeness, availability, adjustment and anchoring, and so forth.
(3) Judgement heuristics are simple strategies/mental processes that people use to find quick solutions to a complex problem by focusing on the most relevant aspects of it.
(4) These heuristics are generally very useful, but they may also lead to severe and systematic errors, or “biases”, because the ensuing judgements are all based on data of limited validity.
(5) Examples of biases are given by the already mentioned over-/under-estimation of probabilities and other fallacies.

Despite individual differences, the programme in (1)–(5) is substantially compatible with Simon’s theory of bounded rationality, that is, rationality is limited in judgements/decisions by the tractability of the problem, the cognitive limitations of the mind, and the time available to make a choice: people thus seek a satisfactory, rather than an optimal, solution [14].

Let us now sketch the most celebrated explanations for conjunctive and disjunctive fallacies.

- Kahneman-Tversky explanation. The heuristics of representativeness is introduced to explain the conjunction fallacy. The event “being a feminist and a bank teller” is judged as more representative than the event “being a bank teller” for Linda’s story [5,9]. This explanation can be criticised, as the heuristics hypothesis works well at an intuitive level, but a general theory of heuristics is missing and different heuristics have to be assumed each time to accommodate different types of fallacies [6,48].
- Misunderstanding explanation. People misunderstand the Linda problem, in the sense that they misinterpret the terms “and” and/or “probability” in the story. This explanation can be criticised, as the fallacy also occurs when the terms “and” and “probability” are not mentioned in the story [48].
- Quantum probability explanation. People judge the sentence “Linda is a feminist and a bank teller” as an ordered sequence, namely, “Linda is a feminist”, then “Linda is a bank teller”. The two questions are incompatible in the standard quantum sense, that is, they produce different statistical distributions when asked in a different order [4,6]. This explanation can be criticised, as new tests seem to confirm that the conjunction fallacy is not directly related to question order effects [49].

Conjunctive and disjunctive fallacies have been identified in several cognitive tests involving Linda-like stories (see, e.g., References [4,6,48,49,50,51]). It should be noted that the original story considered a likely event (L), namely, “Linda is a feminist”, and a unlikely event (U), namely, “Linda is a bank teller”. More recent variants consider the other possible combinations “UU” and “LL”, in both conjunctions and disjunctions. Consider, for example, the following story.

“Bill is 34 years old. He is intelligent, but unimaginative, compulsive and generally lifeless. In college, he was strong in mathematics.”

People were asked in Reference [50] to rank likelihood of the following events.

(U) Bill is a reporter;
(LU) Bill is an accountant and plays jazz for a hobby;
(UU) Bill is a reporter and surfs for a hobby;
(L) Bill works for the Inland Revenue;
(U) Bill plays jazz for a hobby;
(LL) Bill is an accountant and works for the Inland Revenue;
(U) Bill surfs for a hobby;
(L) Bill is an accountant.

Empirical results clearly suggest that conjunctive and disjunctive fallacies are systematically present and do not depend on likelihood of events. Table 1 and Table 2 are illustrative in this direction.

## 4. Decision-Making Errors: The Disjunction Effect

We present in the next two sections examples of type-(ii) errors (cfr., Section 1). Let us preliminarily note that both the disjunction effect, which is discussed in this section and Ellsberg paradox, which will be discussed in Section 5, entail a violation of a fundamental axiom of EUT, namely, the “sure-thing principle”.

The sure-thing principle was formulated by Savage within his subjective EUT (SEUT) formulation [2]. He was inspired by the following story.

“A businessman contemplates buying a certain piece of property. He considers the outcome of the next presidential election relevant. So, […], he asks whether he would buy if he knew that the Democratic candidate were going to win, and decides that he would. Similarly, he considers whether he would buy if he knew that the Republican candidate were going to win, and again finds that he would. Seeing that he would buy in either event, he decides that he should buy, even though he does not know which event obtains […].”

Tversky and Shafir performed a test on the sure-thing principle in which they asked a sample of participants to bet on a gamble that can be played twice, that is a “two-stage gamble” [11]. Let us consider a gamble that has probability 0.5 of winning $200 and probability 0.5 of losing $100. At each stage of the test, respondents were asked whether they wanted to play the gamble. They were presented the following three situations.

(i) Respondents knew they had won the first gamble.
(ii) Respondents knew they had lost the first gamble.
(iii) Respondents did not know the outcome of the first gamble.

According to the sure-thing principle, if respondents decide to play again in both situation (i) and situation (ii), then they should play again also in situation (iii). Tversky and Shafir instead found that:

(1) 69% of the respondents who knew they had won the first gamble decided to play again.
(2) 59% of the respondents who knew they had lost the first gamble decided to play again.
(3) 36% of the respondents who did not know the outcome of the first gamble decided to play again.

The two-stage gamble test violates the sure-thing principle. Moreover, it also violates the law of total probability of Kolmogorovian probability. Indeed, let us denote by $p(E_{P})$ the probability that the respondent plays again without knowing the outcome of the first gamble, by $p(E_{W})$ the probability that the respondent wins the first gamble, by $p(E_{L})$ the probability that the respondent loses the first gamble, by $p(E_{P}|E_{W})$ the conditional probability that the respondent plays again knowing they have won the first gamble, and by $p(E_{P}|E_{L})$ the conditional probability that the respondent plays again knowing they have lost the first gamble. Then, one should have

(18) $$p\left(E_{P}\right)=p\left(E_{W}\right)p\left(E_{P}|E_{W}\right)+p\left(E_{L}\right)p\left(E_{P}|E_{L}\right),$$

according to the law of total probability (see Equation 3, Section 1). But, it is easy to show that one cannot find Kolmogorovian probabilities $p(E_{W})$ and $p\left(E_{L}\right)=1-p\left(E_{W}\right)$ such that the data in Reference [11] are reproduced, that is, $p(E_{P})=0.36$, $p(E_{P}|E_{W})=0.69$ and $p(E_{P}|E_{L})=0.59$, and the law of total probability is satisfied. This deviation from classicality is known as the “disjunction effect”. A similar result was obtained in another test of the sure-thing principle, the “Hawaii problem” [11], and also the “prisoner’s dilemma” can be formulated as a disjunction effect [52,53]. Tversky and Shafir explained the effect suggesting that people are generally “averse to uncertainty”, that is, they prefer sure over unsure choices. In the two-stage gamble, participants prefer to play again in both cases where they have certainty about the outcome of the first gamble, while they refuse to play again when they do not know the outcome of the first gamble.

Recent two-stage gamble have found an empirical pattern that substantially agrees with Tversky-Shafir’s findings [52,53] (see Table 3).

Tversky-Shafir’s explanation is that people are generally “averse to uncertainty”, that is, they prefer sure over unsure actions. In the two-stage gamble test, participants specifically prefer to play again in both cases where they have certainty about the outcome of the first gamble, while they refuse to play again when they do not know the outcome of the first gamble.

## 5. Decision-Making Errors: Ellsberg-Type Paradoxes

EUT is the prevalent theory of DM under uncertainty. Because of its intuitive character, mathematical simplicity and predictive success, EUT has been widely applied to a wide range of choice problems in economics, finance, management and also medicine. The main tenet of EUT is that, in situations of uncertainty individual agents (or, decision-makers) choose in such a way to maximize their utility, or degree of satisfaction.

In the 1940s, von Neumann and Morgenstern proposed a set of “reasonable” axioms on human preferences which allow to uniquely represent uncertain gambles (or, lotteries) by means of a suitable expected utility (EU) functional [54]. However, this formulation only deals with the uncertainty that can be formalized by known probabilities (“objective uncertainty”, or risk). On the other side, situations frequently occur in social science where uncertainty cannot be formalized by known probabilities (“subjective uncertainty, or “ambiguity”) [55]. The so-called “Bayesian paradigm” minimizes the distinction introducing the notion of “subjective probability”: even when probabilities are not known, people may still form their own beliefs (or, priors), and they would aim to maximize EU with respect to these priors [56]. And, indeed, Savage presented in the 1950s an axiomatic formulation of EUT which extends von Neumann and Morgenstern’s to subjective uncertainty (Savage’s SEUT, cfr., Section 4) [2].

Let us provide a simplified presentation of SEUT. Let S be a (discrete, finite) set of all physical states of nature, let $\left\{E_{1},\ldots ,E_{n}\right\}$ be a family of mutually exclusive and exhaustive elementary events, that is, for every i,j=1,…,n, i≠j, $E_{i}=\left\{s_{i}\right\}\subseteq S$, $E_{i}\cap E_{j}=\emptyset$, and $\cup_{i=1}^{n}E_{i}=S$. For every i=1,…,n, let $p(E_{i})$ be the (subjective) probability that $E_{i}$ occurs and suppose that *p* is a Kolmogorovian probability measure. Then, let $\left\{x_{1},\ldots ,x_{n}\right\}$ be a family of monetary payoffs (payoffs are special cases of consequences in SEUT). For every i=1,…,n, let $u(x_{i})$ be the utility value of $x_{i}$ (*u* is a risk-dependent continuous and strictly increasing utility function in SEUT). Finally, let $f=(E_{1},x_{1};\ldots ,E_{n},x_{n})$ define an “act”. Then, the EU of *f* with respect to the Kolmogorovian probability measure *p* is defined as

(19) $$W\left(f\right)=\sum_{i=1}^{n}p\left(E_{i}\right)u\left(x_{i}\right).$$

Savage proved that, if the set F of all acts satisfies a number of “reasonable” axioms, including the sure-thing principle, then, for every f,g∈F, a single Kolmogorovian probability measure *p* and a single (up to positive affine transformations) utility function *u* exist such that *f* is preferred to *g* (or, f≿g) if and only if W(f)≥W(g) [2].

Savage’s theorem is compelling from a normative point of view and testable from a descriptive point of view. Regarding the former, if decision-makers are “rational”, in the sense that their decisions reveal axioms satisfying preferences, then they must all behave as if they maximized an EU with respect to a single Kolmogorovian probability measure interpreted as their subjective probability. Regarding the latter, the validity of SEUT and its underlying axioms can be concretely tested. And, indeed, deviations from classicality have occurred in various DM tests. More specifically, Daniel Ellsberg proved in two seminal thought experiments, the “three-color example” and the “two-urn example”, that decision-makers violate the predictions of SEUT, because they prefer in general acts with known probabilities over acts with unknown probabilities, instead of maximizing EU [12].

Let us start by the three-color example. One urn contains 30 red balls and 60 balls that are either yellow or black, the latter in unknown proportion. One ball will be drawn at random from the urn. Then, a person is asked to bet on pairs of the acts $f_{1}$, $f_{2}$, $f_{3}$ and $f_{4}$ in Table 4.

Ellsberg suggested that most individuals will choose $f_{1}$, revealing the preference $f_{1}≻f_{2}$, and $f_{4}$, revealing the preference $f_{4}≻f_{3}$. This is intuitively reasonable, as $f_{1}$ and $f_{4}$ are unambiguous acts, that is, are associated with events over known probabilities, whereas $f_{2}$ and $f_{3}$ are ambiguous acts, that is, are associated with events over unknown probabilities. This attitude of decision-makers to prefer known over unknown probabilities is called “ambiguity aversion” [12].

Ellsberg preferences above violate the sure-thing principle of SEUT, because the latter predicts consistency of preferences, that is, $f_{1}≻f_{2}$ if and only if $f_{3}≻f_{4}$. Indeed, if we denote by $p(E_{R})=1/3$, $p(E_{Y})$ and $p(E_{B})$ the Kolmogorovian probabilities associated with the events “a red ball is drawn”, “a yellow ball is drawn” and “a black ball is drawn”, respectively, then the EUs are such that $f_{1}≻f_{2}$ if and only if $W\left(f_{1}\right)>W\left(f_{2}\right)$ if and only if $\left(1/3-p\left(E_{B}\right)\right)\left(u\left(100\right)-u\left(0\right)\right)>0$ if and only if $W\left(f_{3}\right)>W\left(f_{4}\right)$ if and only if $f_{3}≻f_{4}$. Equivalently, no assignment of Kolmogorovian probabilities $p(E_{R})=1/3$, $p(E_{Y})$ and $p(E_{B})$ reproduces Ellsberg preferences $f_{1}≻f_{2}$ and $f_{4}≻f_{3}$, with $W\left(f_{1}\right)>W\left(f_{2}\right)$ and $W\left(f_{4}\right)>W\left(f_{3}\right)$, respectively, whence the “Ellsberg paradox”.

Tests on simple urns, but also on financial, management, insurance and medical choices, generally confirm Ellsberg preferences, in most cases indicating ambiguity aversion attitudes (ambiguity seeking attitudes have been identified in a few cases; for a review of empirical studies, see, for example, References [57,58]). A recent test on the three-color example, performed by ourselves, has found a rate of 0.82 for the preference $f_{1}≻f_{2}$ and a rate of 0.78 for the preference $f_{4}≻f_{3}$. The test thus agrees with empirical patterns found in the literature [59].

Various extensions of SEUT have been elaborated to cope with the Ellsberg paradox and non-neutral attitudes towards ambiguity. These “non-EU models” in general weaken the sure-thing principle and explicitly incorporate ambiguity in their formulations. Major models include Choquet EU, cumulative prospect theory, max-min EU, α-max min EU, variational preferences, robust control, and second order beliefs (for a review of theoretical studies, see, for example, References [58,60] and references therein).

The models above accommodate the Ellsberg paradox and have manifold applications in social science. More important, they already depart from the assumption that only Kolmogorovian probabilities can represent subjective priors. However, Mark Machina has recently proved that major non-EU models cannot reproduce his hypothesized preferences in two thought experiments, namely, the “50/51 example” and the “reflection example” [61,62]. Some DM tests have confirmed Machina preferences against the predictions of the non-EU models above [59,63].

The situation becomes even more problematical if one considers the Ellsberg two-urn example, which constitutes the paradigm of real life tests involving managerial and medical decisions. We briefly review the two-urn example and its consequences in the following.

Consider two urns, “urn I” with 100 balls that are either red or black in unknown proportion, and “urn II” exactly with 50 red balls and 50 black balls. One ball is to be drawn at random from each urn. Then, free of charge, a person is asked to bet on pairs of the acts $f_{1}$, $f_{2}$, $f_{3}$ and $f_{4}$ in Table 5.

In this case, acts $f_{2}$ and $f_{4}$ are unambiguous, whereas acts $f_{1}$ and $f_{3}$ are ambiguous. Thus, Ellsberg suggested that, while decision-makers will be generally indifferent between $f_{1}$ and $f_{3}$ and between $f_{2}$ and $f_{4}$, but they will prefer $f_{2}$ over $f_{1}$ and $f_{4}$ over $f_{3}$, thus revealing preferences $f_{2}≻f_{1}$ and $f_{4}≻f_{3}$, together with an ambiguity aversion attitude.

The predictions of SEUT are incompatible with the Ellsberg preferences above. Indeed, if we we denote by $p(E_{R})$ and $p\left(E_{B}\right)=1-p\left(E_{B}\right)$ the Kolmogorovian probabilities associated with the events “a red ball is drawn from Urn I” and “a black ball is drawn from Urn I”, respectively, then the EUs are such that $f_{2}≻f_{1}$ if and only if $W\left(f_{2}\right)>W\left(f_{1}\right)$ if and only if $\left(p\left(E_{R}\right)-1/2\right)\left(u\left(100\right)-u\left(0\right)\right)<0$ if and only if $W\left(f_{3}\right)>W\left(f_{4}\right)$ if and only if $f_{3}≻f_{4}$. Tests on the two-urn example generally confirm Ellsberg preferences, hence an ambiguity aversion attitude of decision-makers. We have tested the two-urn example too, finding a rate of 0.82 for the preference $f_{2}≻f_{1}$ and a rate of 0.84 for the preference $f_{4}≻f_{3}$. The test thus agrees with empirical patterns found in the literature [59].

In each bet, the two-urn example consists in comparing a risky option (Urn II) with an ambiguous option (Urn I). This is the typical scenario one finds in real life tests involving financial, managerial and medical decisions (see, e.g., References [64,65]). One observes in these tests a shift from ambiguity averse to ambiguity seeking attitudes, and viceversa, as due to the presence of “hope and fear effects”. In these situations, indeed, a benchmark value is set which determines whether the decision leads to a “gain”, for example, a better than expected return on an investment, or a “loss”, for example, a failure in a medical treatment, and intuition suggests that the level of probability will play a crucial role in the final decision: if the probability of a gain (loss) is high, then a fear effect (hope effect) occurs in which people tend to be ambiguity averse (seeking). But, as the probability of a gain (loss) decreases, people tend to be less ambiguity averse (seeking), reaching a crossover point in which they become ambiguity seeking (averse), which indicates a shift from a fear (hope) to a hope (fear) effect [64,65].

Shifts of ambiguity attitudes, as due to hope and fear effects, cannot be explained, either from the point of view of traditional SEUT nor from the point of view of more general non-EU models.

The consequence of the empirical findings summarized in this section is that, according to many authors, a unified theory of human choices under uncertainty is still missing (see, e.g., Reference [66]).

## 6. Elaboration of a SCoP Formalism for Cognitive Domains

As anticipated in Section 1, the quantum cognition research programme rests on the recognition that complex cognitive phenomena, like judgements and decisions, exhibit peculiar aspects which prevent in general the application of traditional modelling techniques based on classical set-theoretic formalisms. In particular, the following features have arisen from the phenomena studied in Section 2, Section 3, Section 4 and Section 5.

(i) Judgements and decisions are intrinsically and unavoidably probabilistic processes.
(ii) Judgements and decisions involve constructive processes which create rather than record.
(iii) In these processes, context plays a fundamental role in determining the final response among a range of possible alternatives.
(iv) Different and mutually exclusive alternatives generally disturb each other.
(v) The unrestricted validity classical (Boolean) logic and (Kolmogorovian) probability cannot be assumed in these cases.

These and other considerations have lead several scholars to start using systematically the formalism of quantum theory in Hilbert space, as pure mathematical formalism detached from its physical interpretation, in these classically problematical cognitive phenomena (see, e.g., References [36,37] and references therein).

In this section, we aim to present a unified view on how and why the mathematical formalism of quantum theory is able to provide a general theoretical framework for human cognition. In other words, we aim to provide a foundation of human cognition which justifies the use of quantum theory as a general theory for it. The starting point of our research was the recognition that, in view of points (i)–(v), the systems (or, entities) studied in cognitive domains exhibit deep analogies with quantum entities in the description of what occurs in a laboratory where preparations and measurements are performed.

Indeed, an experiment, or test, on a quantum entity is typically performed in a physics laboratory. The quantum entity preliminarily undergoes a preparation procedure designed by the experimenter, at the end of which “the entity is in a defined state”. This state expresses the “physical reality” of the entity, in the sense that, as a consequence of being in that state, the entity has some “actual” properties independently of any measurement that can be performed on it. Then, when a measurement is performed on the quantum entity, the macroscopic apparatus operates as a measurement context which interacts, on a physical level, with the entity and changes its state in a way that is generally indeterministic, that is, neither controllable nor predictable. Then, the quantum entity, its states, contexts, properties, and the mutual statistical relations are represented in the Hilbert space formalism.

Similarly, a cognitive test, or measurement, is typically performed in a “psychology laboratory”, that is, a spatio-temporal domain where participants give their responses. Suppose, for example, that a judgement/decision test is performed in which a sample of participants have to write down their responses on a paper picking them from a list on a structured questionnaire. The information contained in the questionnaire and the meaning content of the situation that is the object of the decision (literally, what is written in the questionnaire and which has to be judged/decided on) define a preparation procedure for a “conceptual entity”, at the end of which we can say that “the entity is in a defined state”. Thus, a preparation does take place when a cognitive test is performed and each participant “is confronted with this one and unique state”, independently of any belief the participant has about it, because the state was prepared by the experimenter designing the test, before and independently of any individual participating in the test. This state has a conceptual, rather than a physical, nature, but it is a “state of affairs”, because it expresses the meaning content of the questionnaire that was prepared by the experimenter. As such, it is not a mental state or a state of belief. In addition, this state is independent of any operation that can be performed on the entity, hence it expresses the “conceptual reality” of the entity at a given time.

The state of a conceptual entity can change under the effect of the surrounding context, which has in general a cognitive nature and may include the individual participating in the test. Indeed, when the test is performed and a participant is asked to express a judgement or make a decision on the questionnaire, the participant operates as a context which interacts, on a cognitive level, with the entity and changes its state in a way that is generally indeterministic, that is, neither controllable nor predictable. More specifically, when the first participant enters the laboratory and fills out the questionnaire, the participant “interacts with this conceptual state presented to her/him in the test”, but prepared, for example, on a paper, by the experimenter, and changes this state. When the second participant enters the laboratory and fills out the questionnaire, the participant again interacts with this independently prepared conceptual state and contextually changes it, and so on – different individuals may determine different changes of state. As a consequence of this “(measurement) context-induced actualization of potential”, a statistics of responses can be collected, which can be interpreted as outcome probabilities in the large number limit. In addition, some properties of the conceptual entity, which were actual (potential) in the initial state in which the entity is originally prepared, will become potential (actual) in the final state, as a consequence of this contextual interaction.

Now, research on the foundations of quantum physics and quantum probability has allowed to recover the Hilbert space formalism of quantum theory from “operationally reasonable” axioms, resting on well defined empirical notions, directly connected with the operations that are performed in a physics laboratory. In particular, one line of research, initiated by Jauch [67] and Piron [68] in Geneva, and further developed by the research team in Brussels (see, e.g., References [36,37]), has produced a “realistic” and “operational” foundation of this kind, which was called the “State Context Property” (SCoP) formalism. In the ScoP formalism, any physical entity is described by means of the basic notions of “state,” “context,” “property,” and their mutual statistical relations. If suitable axioms are imposed on the mathematical structure underlying the SCoP formalism, then the Hilbert space structure of quantum theory emerges as a unique mathematical representation, up to isomorphisms (see, e.g., References [36,67,68]).

Then, following the analogies above between quantum and conceptual entities, we have elaborated a realistic-operational foundation of conceptual entities, in which any entity of this kind is described in terms of its states, contexts, properties, and statistics of outcomes [69]. We resume and deepen the SCoP description of a conceptual entity in the following.

Let Ω be a conceptual entity, which can be a concept, a combination of concepts, a proposition, or a more complex DM entity. In the SCoP formalism, Ω is described by the 5-tuple (Σ,M,L,μ,ν), where:

(1) Σ is the set of all states of Ω. A state p∈Σ is the result of a preparation procedure of Ω at the end of which Ω is in the state *p*.
(2) M is the set of all contexts of Ω. A context e∈M has typically a cognitive nature and interacts with Ω changing in general its state.
(3) L is the set of all properties of Ω. A property a∈L can be either actual or potential for Ω, depending on its state.
(4) μ is the “state-transition probability function”, that is, an application μ:Σ×M×Σ⟶[0,1], such that, for every p,q∈Σ, e∈M, μ(q,e,p) is the probability that the state *p* of Ω is changed to the state *q* by the context *e*.
(5) ν is the “property-applicability probability function”, that is, an application ν:Σ×L⟶[0,1], such that, for every p,∈Σ, a∈L, ν(p,a) is the probability that the property *a* is actual in the state *p* of Ω.

The above realistic-operational description of any conceptual entity involved in a judgement or a decision in the SCoP formalism suggest representing these entities, states, contexts, properties, probabilities and dynamics within the Hilbert space formalism, in the sense that, in the modelling of a given phenomenon in the cognitive domain, once one identifies the conceptual entity, its states, contexts, properties and statistics of responses, one represents them by means of the usual representation in Hilbert space.

Before coming to the applications, we stress that this description of a judgement/decision process differs from that of other quantum-based approaches to cognition, where the state corresponds to a “mental state of the individual participating in the test” (see, e.g., References [4,6,25]). While the latter description is frequently used, we believe that it does not completely capture all elements involved in such processes, namely, preparation, contextual interaction, state change. In particular, the notion of “state of a conceptual entity” introduces a new element, not directly related to beliefs and not previously used in cognitive science, at the best of our knowledge. The notion is mainly borrowed from physics, as a test in cognitive science, like an experiment in physics, is a bridge between a preparation and a measurement. We believe that this notion of state should be a constitutive element of any statistical theory. This is why we prefer to adopt the description presented here, as it also adheres more closely to the interpretation of the Hilbert space formalism expounded in modern manuals of quantum theory (see, e.g., Reference [21]).

In the following, we will specify the SCoP formalism for probability judgement tests, like those performed on conceptual combinations (Section 6.1), and for DM tests, like those performed on Ellsberg-type situations (Section 6.2), and sketch how a Hilbert space representation of these phenomena can be constructed.

### 6.1. Application of the SCoP Formalism to Human Probability Judgements

We apply in this section the SCoP formalism to the judgements that are expressed when participants are asked about typicality and membership probabilities with respect to concepts and their combinations. This will allow us to construct a general Hilbert space representation of the phenomenology encountered in Section 2.

Let *A* be a concept and let us consider judgement tests in which a sample of participants are asked in a questionnaire to judge typicality or membership of specific items with respect to the concept *A*, as in Section 2. We associate *A* with the conceptual entity $\Omega_{A}$ following the prescriptions in Section 6. In particular, the information and meaning content of the questionnaire provides a preparation of $\Omega_{A}$ at the end of which $\Omega_{A}$ is in a defined state $p_{A}$.

A context *e* interacts with the conceptual entity $\Omega_{A}$ and can change its initial state $p_{A}$. A relevant context is the one introduced by the judgement test itself. Indeed, when the test starts, an interaction occurs between the conceptual entity $\Omega_{A}$ and each participant, in which the state $p_{A}$ of $\Omega_{A}$ generally changes, being transformed into another state *p*. This interaction, which occurs at a cognitive level, as we have seen in Section 6, is specifically described by a measurement context. For example, when in a typicality judgement each participant is asked to pick the most typical item in the family $\{X_{1}$, …, $X_{n}\}$ of *n* distinct items and the participant chooses the item $X_{i}$, i=1,…,n, then the initial state $p_{A}$ of $\Omega_{A}$ is transformed by the typicality measurement context $e_{T}$ into $p_{X_{i}}$, as a consequence of the contextual interaction between $\Omega_{A}$ in $p_{A}$ and $e_{T}$.

The change of state of $\Omega_{A}$ due to a context *e* may be either “deterministic”, hence in principle predictable under the assumption that both $p_{A}$ and *e* are perfectly known, or “intrinsically probabilistic”, that is, only the state-transition probability $\mu (p,e,p_{A})$ that the state $p_{A}$ of $\Omega_{A}$ changes to the state *p* is known. Specifically, in the typicality judgement test above, the typicality of the item $X_{i}$ with respect to the concept *A* is formalized by means of a state-transition probability $\mu (p_{X_{i}},e_{T},p_{A})$, where $e_{T}$ is the typicality measurement context and $p_{X_{i}}$ is the final state of $\Omega_{A}$ at the end of the interaction with $e_{T}$.

Let us now consider a membership judgement test in which each participant is asked to judge membership of the item *X* with respect to the concept *A*. In the SCoP formalism, such test corresponds to a measurement with only two outcomes, or “yes-no measurement”, performed by means of a membership measurement context $e_{M}$, in which a property $a_{X}$ of the conceptual entity $\Omega_{A}$ is tested. Hence, the probability of membership, or membership weight, of the item *X* with respect to the concept *A* is formalized by means of a property-applicability probability $\nu (p_{A},a_{X})$.

Let us now come to the representation in Hilbert space and start by a membership judgement test. The conceptual entity $\Omega_{A}$ is associated with an abstract Hilbert space H, and the initial state $p_{A}$ of *A* is represented by a unit vector |A〉∈H. The membership measurement context $e_{M}$ is represented by a hermitian operator or, equivalently, by a spectral family {M,1−M}, where *M* is an orthogonal projection operator over H and 1 is the identity operator. Then, the membership weight μ(A) of *X* with respect to *A* coincides with the probability that the outcome “yes” is obtained in the interaction of $\Omega_{A}$ in the state $p_{A}$ with $e_{M}$, and is calculated through the Born rule of quantum probability, that is, $\mu \left(A\right)={∥M|A\rangle ∥}^{2}$.

The Born rule also applies to measurements with more than two outcomes. For example, in a typicality judgement test involving the typicality of a family $\{X_{1}$, …, $X_{n}\}$ of *n* distinct items with respect to the concept *A*, the typicality measurement context $e_{T}$ is represented by the spectral measure $\left\{M_{1},\ldots ,M_{n}\right\}$, where $\sum_{i=1}^{n}M_{i}=1$ and $M_{i}M_{j}=\delta_{ij}M_{i}$, i,j=1,…,n, and the probability $\mu_{i}\left(A\right)$ that the item $X_{i}$ is judged as a typical example of the concept *A* is given by the quantum probability $\mu_{i}\left(A\right)={∥M_{i}|A\rangle ∥}^{2}$, i=1,…,n.

An interesting aspect concerns the final state of a conceptual entity $\Omega_{A}$ after a judgement test. We assume the existence of a non-empty class of cognitive tests that correspond to ideal first kind measurements in the standard quantum sense, that is, measurements that satisfy the “Lüders postulate”. Suppose for example that the item $X_{i}$, i=1,…,n, was judged as a typical example of the concept *A* in the typicality test above. Then, we assume that the final state $p_{X_{i}}$ of $\Omega_{A}$ immediately after the interaction with the typicality measurement context $e_{T}$ is represented by the unit vector $|A_{i}\rangle =\frac{M_{i}|A\rangle }{∥M_{i}|A\rangle ∥}$. Of course, we agree that cognitive tests exist which do not correspond to ideal first kind measurements of quantum theory. Nevertheless, it is reasonable to assume that such an ideal first kind state transition occurs at least in some cases, like the ones discussed here [69].

Coming to a more concrete Hilbert space construction, in a judgement test with *n* distinct outcomes, we associate $\Omega_{A}$ with a *n*-dimensional complex Hilbert space H, which can be chosen to be (isomorphic to) the space $C^{n}$, that is, the Hilbert space of all ordered triples of complex numbers. Then, the states of $\Omega_{A}$ are represented by unit vectors of $C^{n}$ and each orthogonal projection operator $M_{i}$ projects onto the one-dimensional subspace generated by the *i*-th vector of the canonical orthonormal (ON) basis of $C^{n}$, i=1,…,n.

The mathematical construction we have put forward here provides a general framework allowing in principle to represent any probability judgement test in Hilbert space. We will apply the present quantum-theoretic framework to over- and under-extension effects and conjunctive and disjunctive fallacies and the disjunction effect in Section 7, Section 7.1 and Section 7.2, respectively.

### 6.2. Application of the SCoP Formalism to Human Decisions

We apply in this section the realistic-operational description of a conceptual entity to the DM tests studied in Section 5 and sketch how a Hilbert space representation can be constructed in these cases.

Let us consider a decision problem where *n* mutually exclusive and exhaustive elementary events, $E_{1},\ldots ,E_{n}$ may occur and the decision-maker has to take a decision between pairs of acts giving different payoffs which depend on the occurrence of $E_{1},\ldots ,E_{n}$. If the required decision has to be taken in the form of a written answer on a questionnaire, a story is preliminarily presented to each individual participating in the test. What each individual reads in the questionnaire, interacts with and decides on defines a preparation of a conceptual entity, which we call “DM entity”, $\Omega_{DM}$.

A state *p* of $\Omega_{DM}$ has a conceptual, not physical, nature and incorporates aspects of uncertainty (e.g., ambiguity). A context *e* does not pertain to $\Omega_{DM}$ but can interact with it. The interaction of $\Omega_{DM}$ with a context *e* may determine a change of the state of $\Omega_{DM}$ from *p* to a different state *q*. The probability of such a state transition is given by the state-transition probability μ(q,e,p), in agreement with the prescriptions in Section 6. We can also complete the SCoP description of $\Omega_{DM}$ introducing properties and property-applicability probabilities, which is not relevant to Ellsberg-type situations but may play a role in more complex decision problems.

A first cognitive context $e_{C}$ relevant to $\Omega_{DM}$ is the context associated with the the *n* mutually exclusive and exhaustive elementary events $E_{1},\ldots ,E_{n}$. It corresponds to a measurement that can be performed on $\Omega_{DM}$ in a given state *p* and has *n* distinct outcomes, or eigenvalues. Each eigenvalue is associated with a final state $p_{i}$ of $\Omega_{DM}$, which is an eigenstate of $e_{C}$, as it satisfies the condition $\mu (p_{i},e_{C},p_{i})=1$. Thus, the context $e_{C}$ is connected with the elementary event $E_{i}$ by the fact that the (subjective) probability $\mu (E_{i},p)$ that the event $E_{i}$ occurs when the conceptual entity $\Omega_{DM}$ is in the state *p* is formalized by means of the state-transition probability $\mu (p_{i},e_{C},p)$, i=1,…,n. Then, in analogy with SEUT, we can define an act $f=(E_{1},x_{1};\ldots ,E_{n},x_{n})$ by associating the monetary payoff $x_{i}$ to the event $E_{i}$, i=1,…,n.

Let us now come to the realistic-operational description of the DM process and consider a decision between acts $f_{1}$ and $f_{2}$ and a decision between acts $f_{3}$ and $f_{4}$ in Table 4, Section 5, for the sake of simplicity. Suppose that the meaning content of the questionnaire has prepared the conceptual entity $\Omega_{DM}$ in the initial state $p_{0}$. Whenever an individual starts pondering between $f_{1}$ and $f_{2}$, before a decision is taken, the individual’s attitude towards ambiguity, for example, ambiguity aversion, is described as a new cognitive context $e_{12}$ operating on $\Omega_{DM}$ in the initial state $p_{0}$ and changing $p_{0}$ to a new state $p_{12}$. The (subjective) probability that the event $E_{i}$ occurs in the state $p_{12}$ is thus $\mu \left(E_{i},p_{12}\right)=\mu \left(p_{i},e_{C},p_{12}\right)$, i=1,…,n. Similarly, whenever the individual starts pondering between $f_{3}$ and $f_{4}$, before a decision is taken, the individual’s attitude towards ambiguity, for example, again ambiguity aversion, is described as another cognitive context $e_{34}$ operating on $\Omega_{DM}$ in the initial state $p_{0}$ and changing $p_{0}$ to a new state $p_{34}$. The (subjective) probability that the event $E_{i}$ occurs in the state $p_{34}$ is thus $\mu \left(E_{i},p_{34}\right)=\mu \left(p_{i},e_{C},p_{34}\right)$, i=1,…,n. The ambiguity averse final states $p_{34}$ and $p_{34}$ are responsible of the “inversion of preferences” which occur in Ellsberg-type situations [70].

Finally, the actual decision between acts $f_{1}$ and $f_{2}$ is operationally described as a decision measurement context $e_{D_{1}}$ acting on $\Omega_{DM}$ in the ambiguity averse state $p_{12}$, with possible outcomes “yes” and “no”. Similarly, the actual decision between acts $f_{3}$ and $f_{4}$ is operationally described as a decision measurement context $e_{D_{2}}$ acting on $\Omega_{DM}$ in the ambiguity averse state $p_{34}$, with possible outcomes “yes” and “no”. These contexts give rise of the statistics of outcomes in Ellsberg-type DM tests.

The representation of the DM situation above is straightforward. The DM entity $\Omega_{DM}$ is associated with an abstract Hilbert space H. The presence of *n* mutually exclusive and exhaustive elementary events suggests choosing H in such a way that it is (isomorphic to) the the complex Hilbert space $C^{n}$. Hence, a state $p_{v}$ of $\Omega_{DM}$ is represented by a unit vector $|v\rangle \in C^{n}$. A context *e* is instead represented by a hermitian operator or, equivalently, by a spectral family, over $C^{n}$. Thus, the context $e_{C}$ above is represented by the spectral family $\left\{P_{1},\ldots ,P_{n}\right\}$, where each $P_{i}$ represents the elementary event $E_{i}$ and projects onto the one-dimensional subspace generated by the *i*-th vector of the canonical ON basis of $C^{n}$. Then, the (subjective) probability $\mu (p,E_{i})$ that the event $E_{i}$, represented by the orthogonal projection operator $P_{i}$, occurs when $\Omega_{DM}$ is in the state $p_{v}$, represented by the unit vector |v〉, is obtained through the Born rule of quantum probability, namely, $\mu \left(p,E_{i}\right)={∥P_{i}|v\rangle ∥}^{2}$. An act $f=(E_{1},x_{1};\ldots ,E_{n},x_{n})$ is represented by the hermitian operator $F=\sum_{i=1}^{n}u\left(x_{i}\right)P_{i}$, where $u(x_{i})$ is the utility value associated with the payoff $x_{i}$. This suggests to finally introduce a state-dependent EU functional $W_{v}\left(f\right)=\langle v|F|v\rangle$.

The mathematical construction we have put forward here provides a general framework allowing in principle to represent any DM test in Hilbert space. We will apply the present quantum-theoretic framework to the Ellsberg three-color and two-urn examples in Section 8.

## 7. A Quantum Framework to Represent Concepts and Their Combinations

Relying on a one-decade research on the foundations of quantum theory (cfr., Section 6.1), we have developed a novel framework to represent concepts and their combinations. In this framework, a conceptual entity can be expressed in terms of the SCoP formalism, hence it admits a Hilbert space representation [34,71,72]. As we have seen in Section 6, the main novelties can be summarised as follows.

(i) A concept is an entity in a defined state, rather than a container of items.
(ii) A context is a factor that influences the concept and generally changes its state.
(iii) Quantities as typicality, membership, and so forth can be measured on concepts and have different probabilistic values in different states of the concept.

We have applied the mathematical formalism of quantum theory to represent conceptual entities, states, contexts and the corresponding probabilities. The quantum-theoretic framework we have developed successfully models membership weights of concepts and their combinations [22,31,32,34,35], conceptual typicality [34,71,72] and borderline contradictions [73] in Section 2.

We expose now the fundamentals of the quantum-theoretic framework, starting from the conjunction of two concepts. We refer to the membership probability judgement tests in Section 2. Let *X* be an item, let *A* and *B* be two concepts, and let ‘AandB’ be their conjunction. Then, let μ(A), μ(B) and μ(AandB) be the membership weights of *X* with respect to *A*, *B* and ‘AandB’, respectively.

As seen in Section 6.1, conceptual entities are represented in an abstract Hilbert space H. When a membership judgement test is performed and a questionnaire is presented to the participants, the initial state of the concept *A* is represented by the unit vector |A〉∈H, $∥|A\rangle ∥=1$, and the initial state of the concept *B* is represented by the unit vector |B〉∈H, $∥|B\rangle ∥=1$. Suppose that |A〉 and |B〉 are orthogonal, that is, 〈A|B〉=0, for the sake of simplicity. We represent the initial state of the conjunction ‘AandB’ by the unit vector $|A\;\mathrm{and}\;B\rangle =\frac{1}{\sqrt{2}}(|A\rangle +\left|B\rangle \right)$.

The judgement measurement of a person who judges the membership of *X* with respect to *A*, *B* and ‘AandB’ is represented by the orthogonal projection operator *M* over H. We calculate membership weights by means of the Born rule of quantum probability. More precisely,

(20) $$\begin{array}{ccc} \mu (A) & = & \langle A|M|A\rangle , \end{array}$$

(21) $$\begin{array}{ccc} \mu (B) & = & \langle B|M|B\rangle \end{array}$$

are the probabilities of membership, that is, membership weights, while

(22) $$\begin{array}{ccc} 1-\mu (A) & = & \langle A|1-M|A\rangle , \end{array}$$

(23) $$\begin{array}{ccc} 1-\mu (B) & = & \langle B|1-M|B\rangle \end{array}$$

are the probabilities of non-membership, in the initial states of concepts *A* and *B*. The probability of membership in the initial state of ‘AandB’ is thus given by:

(24) μ(AandB)=〈AandB|M|AandB〉=12μ(A)+μ(B)+Re(〈A|M|B〉).

Equation (24) expresses the “quantum probability formula for the conjunction”. Different cases are possible for the interference term for the conjunction Re(〈A|M|B〉), namely,

- Re(〈A|M|B〉)>0 indicates to constructive interference;
- Re(〈A|M|B〉)=0 indicates to absence of interference;
- Re(〈A|M|B〉)<0 indicates to destructive interference.

We follow the same procedure for the disjunction of two concepts. Let μ(A), μ(B) and μ(AorB) be the membership weights of the item *X* with respect to the concepts *A*, *B* and their disjunction ‘AandB’, respectively. When a membership judgement test is performed and a questionnaire is presented to the participants, the initial state of the concept *A* is represented by the unit vector |A〉∈H, and the initial state of the concept *B* is represented by the unit vector |B〉∈H, with 〈A|B〉=0. Then, the initial state of the disjunction ‘AorB’ is represented by the unit vector $|A\;\mathrm{or}\;B\rangle =\frac{1}{\sqrt{2}}(|A\rangle +\left|B\rangle \right)$.

Using the symbols in Equations (20)–(24), we get that the probability of membership, or membership weight, of *X* with respect to ‘AorB’ is given by the following “quantum probability formula for the disjunction”

(25) μ(AorB)=12μ(A)+μ(B)+Re(〈A|M|B〉),

where Re(〈A|M|B〉) is the interference term for the disjunction. The orthogonal projection operator *M* in Equation (24) actually depends on the item *X* and the conjunction ‘and’, whereas the projection operator *M* in Equation (25) actually depends on the item *X* and the disjunction ‘or’. We have omitted such dependence here, which actually makes Equations (24) and (25) different, for the sake of simplicity.

Let us now construct an explicit representation for the conjunction and the disjunction of two concepts in the complex Hilbert space $C^{3}$. Let {(1,0,0),(0,1,0),(0,0,1)} be the canonical ON basis of $C^{3}$ (details can be found in References [22,34,35]).

Let us again start by the conjunction case. Let *X* be an item and let μ(A), μ(B) and μ(AandB) be the membership weights of *X* with respect to *A*, *B* and ‘AandB’, respectively. We introduce the following quantities:

(26) $$\begin{array}{c} \begin{array}{cc} a= & \left\{\begin{array}{cc} 1-\mu (A) & \mathrm{if}\;\mu (A)+\mu (B)\leq 1\\ \mu (A) & \mathrm{if}\;\mu (A)+\mu (B)>1 \end{array}\right.\\ b= & \left\{\begin{array}{cc} 1-\mu (B) & \mathrm{if}\;\mu (A)+\mu (B)\leq 1\\ \mu (B) & \mathrm{if}\;\mu (A)+\mu (B)>1 \end{array}\right.\\ \gamma = & \left\{\begin{array}{cc} 180^{\circ } & \mathrm{if}\;\mu (A)+\mu (B)\leq 1\\ 0 & \mathrm{if}\;\mu (A)+\mu (B)>1 \end{array}\right.. \end{array} \end{array}$$

The quantum probability formula for the conceptual conjunction in Equation (24) becomes

(27) $$\mu \left(A\;\mathrm{and}\;B\right)=\frac{1}{2}\left(\mu (A)+\mu (B)\right)+\sqrt{\left(1-a)(1-b\right)}\cos\theta_{c},$$

where $\theta_{c}$ is the “interference angle for the conjunction”. One can easily verify that the unit vectors

(28) $$\begin{array}{ccc} |A\rangle & = & \left(\sqrt{a},0,\sqrt{1-a}\right), \end{array}$$

(29) $$\begin{array}{ccc} |B\rangle & = & \left\{\begin{array}{cc} e^{i(\theta_{c}+\gamma )}\left(\sqrt{\frac{\left(1-a)(1-b\right)}{a}},\sqrt{\frac{a+b-1}{a}},-\sqrt{1-b}\right) & \mathrm{if}\;\mu (A)\neq 0\\ e^{i(\theta_{c}+\gamma )}\left(0,1,0\right) & \mathrm{if}\;\mu (A)=0 \end{array}\right. \end{array}$$

satisfy Equations (20)–(24). Indeed, if μ(A)+μ(B)>1, these equations are satisfied by choosing the orthogonal projection operator *M* to project onto the subspace generated by the unit vectors (1,0,0) and (0,1,0) of the canonical ON basis of $C^{3}$. If instead μ(A)+μ(B)≤1, the equations are satisfied by choosing *M* to project onto the subspace generated by the unit vector (0,0,1) of the same basis [22].

Let us finally study the disjunction case. Let *X* be an item and let μ(A), μ(B) and μ(AorB) be the membership weights of *X* with respect to *A*, *B* and ‘AorB’, respectively. We introduce the quantities *a*, *b* and γ as in Equation (26). The quantum probability formula for the conceptual disjunction in Equation (25) then becomes

(30) $$\mu \left(A\;\mathrm{or}\;B\right)=\frac{1}{2}\left(\mu (A)+\mu (B)\right)+\sqrt{\left(1-a)(1-b\right)}\cos\theta_{d},$$

where $\theta_{d}$ is the “interference angle for the conjunction”. The unit vectors |A〉 and |B〉 are obtained from Equations (28) and (29) by replacing $\theta_{c}$ with $\theta_{d}$.

The quantum models above enable faithful representation of several data sets, including those in References [7,8]. While a quantum-theoretic framework that models all existing cases of conceptual combinations requires an extension in Fock spaces (see, e.g., References [22,34,74]), the models presented here are anyway falsifiable, as they provide precise predictions on the empirical relationships between membership weights. We focus on conceptual conjunctions, for the sake of brevity. Let us set $\Delta_{AB}=\mu \left(A\;\mathrm{and}\;B\right)-\frac{1}{2}\left(\mu (A)+\mu (B)\right)$ and distinguish two cases, as follows.

If μ(A)+μ(B)≤1, μ(A),μ(B)≠1, then,

(31) $$-\sqrt{\mu (A)\mu (B)}\leq \Delta_{AB}\leq \sqrt{\mu (A)\mu (B)}.$$

If μ(A)+μ(B)>1, μ(A),μ(B)≠1, then,

(32) $$-\sqrt{\left(1-\mu (A))(1-\mu (B)\right)}\leq \Delta_{AB}\leq \sqrt{\left(1-\mu (A))(1-\mu (B)\right)}.$$

Similar constraints have to be satisfied in the disjunction case for the quantum model to be able to represent empirical data.

Let us now consider some relevant examples. In the case of *Razor* with respect to *Weapons*, *Tools* and their conjunction *Weapons and Tools*, in Section (Section 2), the quantum model gives

(33) $$\begin{array}{ccc} \theta_{c} & = & 64.02^{\circ }, \end{array}$$

(34) $$\begin{array}{ccc} |A\rangle & = & (0.79,0,0.61), \end{array}$$

(35) $$\begin{array}{ccc} |B\rangle & = & e^{i64.02^{\circ }}\left(0.36,0.81,-0.47\right). \end{array}$$

that is, the concepts *A* and *B* “constructively interfere” in the conjunction ‘AandB’.

In the case of *Curry* with respect to *Spices*, *Herbs* and their disjunction *Spices or Herbs*, in Section (Section 2), the quantum model gives

(36) $$\begin{array}{ccc} \theta_{d} & = & 65.91^{\circ }, \end{array}$$

(37) $$\begin{array}{ccc} |A\rangle & = & (0.95,0,0.32), \end{array}$$

(38) $$\begin{array}{ccc} |B\rangle & = & e^{i65.91^{\circ }}\left(0.26,0.58,-0.77\right). \end{array}$$

Also in this case, the concepts *A* and *B* “constructively interfere” in the disjunction ‘AorB’.

The quantum model also works in other cases that are classically highly problematical. For example, Hampton measured the membership weights of the item *Mint* with respect to *Food*, *Plant* and their conjunction *Food and Plant*, finding μ(A)=0.87, μ(B)=0.81 and μ(AandB)=0.90, that is, a case of double overextension [7]. These data are modelled by

(39) $$\begin{array}{ccc} \theta_{c} & = & 67.56^{\circ }, \end{array}$$

(40) $$\begin{array}{ccc} |A\rangle & = & (0.93,0,0.36), \end{array}$$

(41) $$\begin{array}{ccc} |B\rangle & = & e^{i67.56^{\circ }}\left(0.17,0.88,-0.44\right). \end{array}$$

Furthermore, Hampton measured the membership weights of *Ashtray* with respect to *Home Furnishing*, *Furniture* and their disjunction *Home Furnishing or Furniture*, finding μ(A)=0.70, μ(B)=0.30 and μ(AorB)=0.25, that is, a case of double underextension [8]. These data are modelled by

(42) $$\begin{array}{ccc} \theta_{d} & = & 123.06^{\circ }, \end{array}$$

(43) $$\begin{array}{ccc} |A\rangle & = & (0.84,0,0.55), \end{array}$$

(44) $$\begin{array}{ccc} |B\rangle & = & e^{i123.06^{\circ }}\left(0.26,0.58,-0.77\right). \end{array}$$

We conclude this section with two remarks which allow us to understand the effectiveness of the quantum-theoretic framework presented here, together with its predictive and explanatory capacity.

Firstly, we have recently generalized Hampton’s tests, extending them to conjunctions and negations of two concepts [74]. More precisely, we have tested membership weights of items with respect to two concepts *A* and *B*, their negations ‘notA’ and ‘notB’, and all possible conjunctions ‘AandB’, ‘AandnotB’, ‘notAandB’, and ‘notAandnotB’. We have found systematic overextension in all conjunctions, cases of double overextension, deviations from classicality in conceptual negations, and a new unexpected and significant deviation from the marginal law. For example, we have found that

(45) μ(AandB)+μ(AandnotB)−μ(A)≈0.5.

Looking at data in Reference [74], the left side of Equation (45) is systematically different from 0, whereas a value of 0 is what one would expect in a Kolmogorovian probability framework. But, the value is also roughly constant and independent of the item and the pair of concepts. This non-classical result is exactly predicted by the Fock space model in References [22,34,74], which thus allows to obtain new classically unexplainable results [75,76].

Secondly, the quantum-theoretic framework presented here reveals the presence of genuine quantum effects in the formation and combination of natural concepts, namely, contextuality, interference, superposition, and emergence. In particular, to grasp the importance of the latter, let two compare the following experimental results in References [8,74] for the item *Olive* with respect to the natural concepts *Fruits*, *Vegetables*, *Fruits and Vegetables*, and *Fruits or Vegetables*. Hampton found μ(A)=0.50, μ(B)=0.10 and μ(AorB)=0.80 in his test [8], whereas we found μ(A)=0.56, μ(B)=0.63 and μ(AandB)=0.65 in ours [74].

Despite individual numerical differences, which can however be explained [74], both data exhibit non-classicality, namely, violation of Equation (16) in the disjunction and double overextension in the conjunction. More important, *Olive* scores similar membership weights in the disjunction and the conjunction case, that is, its membership weight is substantially independent of the combination that is considered. This clearly indicates that situations exist where people do not really take into account whether two concepts are combined through the connective ‘or’ or the connective ‘and’, but they actually judge whether the given item is a member of the new emergent concept, meant as a standalone entity, regardless of the way it is obtained. This is a form of “conceptual emergence” and reveals the presence of a conceptual, or emergent, reasoning, which acts simultaneously with logical reasoning, in human thought [34].

We believe that it is this conceptual, or emergent, reasoning that is responsible of the other probability judgement errors studied in Section 3. In fact, the quantum conceptual framework presented here enables modelling of more general probability judgements, as we will explicitly see in the next two sections.

### 7.1. An Application to Conjunctive and Disjunctive Fallacies

We present in this section the quantum conceptual explanation we have recently elaborated for conjunctive and disjunctive fallacies—the observed empirical deviations from classicality can be interpreted as overextension effects and underextension effects, respectively. Hence, they can modelled using the quantum-theoretic framework we have developed in Section 7 for conceptual combinations. As such, these fallacies are not real errors but, rather, genuine expressions of quantum structures, in human reasoning, namely, conceptual emergence and an underlying conceptual reasoning.

We will now elaborate quantum models for the conjunction and the disjunction fallacy borrowing terminology, methods and the formalism we have developed to model conceptual conjunctions and disjunctions.

We refer to the probability judgement tests in Section 3. Let us denote by *X* the item *Linda*, by *A* the concept *Feminist*, by *B* the concept *Bank Teller*, by ‘AandB’ the conjunction *Feminist and Bank Teller*, and by ‘AorB’ the disjunction *Feminist or Bank Teller*. Moreover, let us denote by μ(A),μ(B),μ(AandB), and μ(AorB), the probability that Linda is judged as feminist, bank teller, feminist and bank teller, and feminist or bank teller, respectively. Finally, we use the terminology of concept theory: thus, μ(A) becomes the membership weight of *Linda* with respect to *Feminist*, μ(B) the membership weight of *Linda* with respect to *Bank Teller*, μ(AandB) the membership weight of *Linda* with respect to *Feminist and Bank Teller*, and μ(AorB) the membership weight of *Linda* with respect to *Feminist or Bank Teller*.

We start by the conjunction fallacy and represent conceptual entities in an abstract Hilbert space H. Concepts *A* and *B* are represented by the mutually orthogonal unit vectors |A〉∈H and |B〉∈H, respectively. The conjunction ‘AandB’ is instead represented by the unit vector $|A\;\mathrm{and}\;B\rangle =\frac{1}{\sqrt{2}}(|A\rangle +\left|B\rangle \right)$. Then, we use the quantum probability formula for the conjunction of two concepts in Equation (24) and rewrite it in the following:

(46) μ(AandB)=12μ(A)+μ(B)+Re(〈A|M|B〉).

In Reference [10], μ(A)+μ(B)=0.83+0.26=1.09>1. Hence, in the $C^{3}$ representation, *M* projects onto the subspace generated by (1,0,0) and (0,1,0), the unit vectors become

(47) $$\begin{array}{ccc} |A\rangle & = & \left(\sqrt{\mu (A)},0,\sqrt{1-\mu (A)}\right), \end{array}$$

(48) $$\begin{array}{ccc} |B\rangle & = & e^{i\theta_{c}}\left(\sqrt{\frac{\left(1-\mu (A))(1-\mu (B)\right)}{\mu (A)}},\sqrt{\frac{\mu (A)+\mu (B)-1}{\mu (A)}},-\sqrt{1-\mu (B)}\right), \end{array}$$

and the quantum probability formula can be written as

(49) $$\mu \left(A\;\mathrm{and}\;B\right)=\frac{1}{2}\left(\mu (A)+\mu (B)\right)+\sqrt{\left(1-\mu (A))(1-\mu (B)\right)}\cos\theta_{c},$$

where $\theta_{c}$ is the interference angle for the conjunction. From the data in Reference [10]:

(50) $$\begin{array}{ccc} \mu (A) & = & 0.83, \end{array}$$

(51) $$\begin{array}{ccc} \mu (B) & = & 0.26, \end{array}$$

(52) $$\begin{array}{ccc} \mu (A\;\mathrm{and}\;B) & = & 0.36, \end{array}$$

we get

(53) $$\begin{array}{ccc} \theta_{c} & = & 121.44^{\circ }, \end{array}$$

(54) $$\begin{array}{ccc} |A\rangle & = & (0.91,0,0.41), \end{array}$$

(55) $$\begin{array}{ccc} |B\rangle & = & e^{i121.44^{\circ }}\left(0.39,0.33,-0.86\right), \end{array}$$

that is, the concepts *A* and *B* “destructively interfere” in the conjunction ‘AandB’.

Let us then come to the disjunction fallacy and follow the same steps we implemented for conceptual disjunction. In the Hilbert space H representation, formulas are the same as in the conjunction fallacy modelling, with the symbol ‘or’ replacing the symbol ‘and’. In the specific $C^{3}$ representation, formulas for the disjunction fallacy are instead obtained by replacing in Equations (48) and (49) the angle $\theta_{c}$ with the interference angle for the disjunction $\theta_{d}$.

From the data in Reference [10]:

(56) $$\begin{array}{ccc} \mu (A) & = & 0.83, \end{array}$$

(57) $$\begin{array}{ccc} \mu (B) & = & 0.26, \end{array}$$

(58) $$\begin{array}{ccc} \mu (A\;\mathrm{or}\;B) & = & 0.60, \end{array}$$

we get

(59) $$\begin{array}{ccc} \theta_{c} & = & 81.08^{\circ }, \end{array}$$

(60) $$\begin{array}{ccc} |A\rangle & = & (0.91,0,0.41), \end{array}$$

(61) $$\begin{array}{ccc} |B\rangle & = & e^{i81.08^{\circ }}\left(0.39,0.33,-0.86\right), \end{array}$$

that is, the concepts *A* and *B* “constructively interfere” in the disjunction ‘AorB’.

The quantum-theoretic framework enables faithful representation of different data sets (see also Table 1 and Table 2 in Section 3). Then, the explanation we propose for conjunctive and disjunctive fallacies is that, when the item *Linda* is considered, together with her story, the concepts *Feminist* and *Bank Teller* destructively interfere in the conjunction *Feminist and Bank Teller*, meant as a newly emerging standalone conceptual entity; the same concepts, instead constructively interfere in the disjunction *Feminist or Bank Teller*, again meant as a newly emerging standalone conceptual entity [34].

Conjunctive and disjunctive fallacies in human probability judgements can be accounted for genuine quantum effects, that is, contextuality, interference, superposition and emergence. The conjunction and the disjunction fallacy in decision theory are respectively the decision-theoretic counterparts of over- and under-extension effects in concept theory. In analogy with the case of double overextension in conceptual conjunctions and double underextension in conceptual disjunctions, the quantum-theoretic framework also predicts the presence of “double conjunction fallacies” and “double disjunction fallacies” [35].

### 7.2. An Application to the Disjunction Effect

We review in this section the quantum conceptual explanation that we have recently elaborated for the disjunction effect—the empirically observed deviations from classicality can be interpreted as underextension effects, hence the disjunction effect can be modelled by using the quantum-theoretic framework we have developed in Section 7 for conceptual disjunctions. In the two-stage gamble, uncertainty aversion is just one, although an important one, of the conceptual landscapes surrounding the decision situation, playing the role of context and determining the final choice. Again, the disjunction effect reveals the presence of genuine quantum structures in human reasoning, namely, contextuality, interference, superposition and emergence.

The quantum-theoretic framework for conceptual disjunctions enables modelling the disjunction effect in both the two-stage gamble and the Hawaii problem, as we have proved in Reference [35]. In this section, we apply it to the two-stage gamble.

We refer to the DM error studied in Section 4. Let us preliminarily introduce the terminology of Section 7 and denote by *A* the conceptual situation of having won the first gamble, by *B* the conceptual situation of having lost lost the first gamble, and by ‘AorB’ the conceptual situation of having won “or” lost the first gamble. Moreover, let us denote by μ(A) the probability of playing again in the conceptual situation *A*, by μ(B) the probability of playing again in the conceptual situation *B*, and by μ(AorB) the probability of playing again in the conceptual situation ‘AorB’. The terminology we use is consistent with the fact that we interpret these decision probabilities as membership weights with respect to conceptual situations and their disjunction.

We represent conceptual entities in an abstract Hilbert space H. Conceptual situations *A* and *B* are represented by the mutually orthogonal unit vectors |A〉∈H and |B〉∈H, respectively, 〈A|B〉=0. The disjunction ‘AorB’ is instead represented by the unit vector $|A\;\mathrm{or}\;B\rangle =\frac{1}{\sqrt{2}}(|A\rangle +\left|B\rangle \right)$. Finally, the decision measurement about playing again is represented by the orthogonal projection operator over H. Then, we apply to the two-stage gamble test the quantum probability formula in for conceptual disjunctions Equation (25) and rewrite it in the following:

(62) μ(AorB)=12μ(A)+μ(B)+Re(〈A|M|B〉).

In Reference [11], μ(A)+μ(B)=0.69+0.59=1.28>1. Hence, in the $C^{3}$ representation, *M* projects onto the subspace generated by (1,0,0) and (0,1,0), and we have

(63) $$\begin{array}{ccc} |A\rangle & = & \left(\sqrt{\mu (A)},0,\sqrt{1-\mu (A)}\right), \end{array}$$

(64) $$\begin{array}{ccc} |B\rangle & = & e^{i\theta_{d}}\left(\sqrt{\frac{\left(1-\mu (A))(1-\mu (B)\right)}{\mu (A)}},\sqrt{\frac{\mu (A)+\mu (B)-1}{\mu (A)}},-\sqrt{1-\mu (B)}\right), \end{array}$$

and the quantum probability formula can be written as

(65) $$\mu \left(A\;\mathrm{or}\;B\right)=\frac{1}{2}\left(\mu (A)+\mu (B)\right)+\sqrt{\left(1-\mu (A))(1-\mu (B)\right)}\cos\theta_{d},$$

where $\theta_{d}$ is the interference angle for the disjunction.

From the data in Reference [11]:

(66) $$\begin{array}{ccc} \mu (A) & = & 0.69, \end{array}$$

(67) $$\begin{array}{ccc} \mu (B) & = & 0.59, \end{array}$$

(68) $$\begin{array}{ccc} \mu (A\;\mathrm{or}\;B) & = & 0.36, \end{array}$$

we get

(69) $$\begin{array}{ccc} \theta_{d} & = & 141.76^{\circ }, \end{array}$$

(70) $$\begin{array}{ccc} |A\rangle & = & (0.83,0,0.56), \end{array}$$

(71) $$\begin{array}{ccc} |B\rangle & = & e^{i141.76^{\circ }}\left(0.43,0.64,-0.64\right), \end{array}$$

that is, the conceptual situations *A* and *B* “destructively interfere” in the disjunction ‘AorB’.

The quantum-theoretic framework enables faithful representation of different data sets (see also Table 3 in Section 4), explaining uncertainty aversion as one of the possible context effect produced by the overall conceptual landscape surrounding the decision situation. In the disjunction effect, a case of destructive interference occurs when the conceptual situations “winning the first gamble” and “losing the first gamble” combine in the conceptual disjunction, which is responsible of the double underextension effects observed empirically. This means that the presence of a conceptual, or emergent, reasoning can be identified in the disjunction effect too.

## 8. A Quantum Framework for Ellsberg-Type Paradoxes

We have recently developed a theoretical framework, along the lines sketched in Section 6.2, that uses the mathematical formalism of quantum theory to model human DM under uncertainty. The quantum-theoretic framework:

(i) provides a unitary solution for a variety of paradoxes of EUT, including Ellsberg and Machina paradoxes [33,77,78];
(ii) enables faithful representation of various sets of empirical data on these paradoxes [33,59,77];
(iii) successfully models shifts of attitudes towards uncertainty, from ambiguity averse to ambiguity seeking, and viceversa, as due to hope and fear effects [79];
(iv) opens the way towards a quantum-based extension of SEUT [29,33,77].

We rely on the quantum conceptual framework in Section 6.2 and assume that, in any DM process, an interaction occurs between the conceptual entity that is the object of the decision, or DM entity, and the cognitive context, which may include the decision-maker. This interaction changes the (conceptual) state of the DM entity.

Let us apply the quantum-theoretic framework to the Ellsberg three-color example presented in Section 5. The DM entity $\Omega_{DM}$ is the urn with 30 red balls and 60 yellow and black balls in unknown proportion, and is associated with the Hilbert space $C^{3}$. Let {(1,0,0),(0,1,0),(0,0,1)} be the canonical ON basis of $C^{3}$. For every i=R,Y,B, the elementary event $E_{i}$ is represented by the one-dimensional orthogonal projection operator $P_{i}=\left|i\rangle \langle i\right|$, where |R〉=(1,0,0), |Y〉=(0,1,0) and |B〉=(0,0,1). A state $p_{v}$ of $\Omega_{DM}$ incorporates aspects of ambiguity and is represented by the unit vector $|v\rangle \in C^{3}$, $∥|v\rangle ∥=1$, that is,

(72) $$|v\rangle =\rho_{R}e^{i\theta_{R}}|R\rangle +\rho_{Y}e^{i\theta_{Y}}|Y\rangle +\rho_{B}e^{i\theta_{B}}|B\rangle =\left(\rho_{R}e^{i\theta_{R}},\rho_{Y}e^{i\theta_{Y}},\rho_{B}e^{i\theta_{B}}\right),$$

where $\rho_{R},\rho_{Y},\rho_{B}\geq 0$, $\rho_{R}^{2}+\rho_{Y}^{2}+\rho_{B}^{2}=1$, $\theta_{R},\theta_{Y},\theta_{B}\in R$

For every i=R,Y,B, the (subjective) probability $\mu_{v}\left(E_{i}\right)$ that $E_{i}$ occurs in the state $p_{v}$ of $\Omega_{DM}$ is obtained through the Born rule of quantum probability, that is,

(73) $$\mu_{v}\left(E_{i}\right)=\langle v|P_{i}\left|v\rangle =\right|\langle i|v\rangle {|}^{2}=\rho_{i}^{2}.$$

Compatibility with the three-color example requires that $\rho_{R}^{2}=\frac{1}{3}$, hence

(74) $$|v\rangle =\left(\frac{1}{\sqrt{3}}e^{i\theta_{R}},\rho_{Y}e^{i\theta_{Y}},\rho_{B}e^{i\theta_{B}}\right)=\left(\frac{1}{\sqrt{3}}e^{i\theta_{R}},\rho_{Y}e^{i\theta_{Y}},\sqrt{\frac{2}{3}-\rho_{y}^{2}}e^{i\theta_{B}}\right).$$

Acts $f_{1}$, $f_{2}$, $f_{3}$ and $f_{4}$ in Table 4 are represented by the hermitian operators

(75) $$\begin{array}{ccc} F_{1} & = & u\left(100\right)P_{R}+u\left(0\right)P_{Y}+u\left(0\right)P_{B}, \end{array}$$

(76) $$\begin{array}{ccc} F_{2} & = & u\left(0\right)P_{R}+u\left(0\right)P_{Y}+u\left(100\right)P_{B}, \end{array}$$

(77) $$\begin{array}{ccc} F_{3} & = & u\left(100\right)P_{R}+u\left(100\right)P_{Y}+u\left(0\right)P_{B}, \end{array}$$

(78) $$\begin{array}{ccc} F_{4} & = & u\left(0\right)P_{R}+u\left(100\right)P_{Y}+u\left(100\right)P_{B}. \end{array}$$

Thus, for every i=1,2,3,4, the EU $W_{v}\left(f_{i}\right)$ of $f_{i}$ in the state $p_{v}$ is given by

(79) $$\begin{array}{ccc} W_{v}\left(f_{1}\right) & = & \langle v|F_{1}|v\rangle =\frac{1}{3}u\left(100\right)+\frac{2}{3}u\left(0\right), \end{array}$$

(80) $$\begin{array}{ccc} W_{v}\left(f_{2}\right) & = & \langle v|F_{2}|v\rangle =\left(\frac{1}{3}+\rho_{Y}^{2}\right)u\left(0\right)+\left(\frac{2}{3}-\rho_{Y}^{2}\right)u\left(100\right), \end{array}$$

(81) $$\begin{array}{ccc} W_{v}\left(f_{3}\right) & = & \langle v|F_{3}|v\rangle =\left(\frac{1}{3}+\rho_{Y}^{2}\right)u\left(100\right)+\left(\frac{2}{3}-\rho_{Y}^{2}\right)u\left(0\right), \end{array}$$

(82) $$\begin{array}{ccc} W_{v}\left(f_{4}\right) & = & \langle v|F_{4}|v\rangle =\frac{1}{3}u\left(0\right)+\frac{2}{3}u\left(100\right) \end{array}$$

As we can see, the EUs $W_{v}\left(f_{1}\right)$ and $W_{v}\left(f_{4}\right)$ do not depend on $p_{v}$, in agreement with the fact that $f_{1}$ and $f_{4}$ are unambiguous acts. On the contrary, the EUs $W_{v}\left(f_{2}\right)$ and $W_{v}\left(f_{3}\right)$ do depend on $p_{v}$, in agreement with the fact that $f_{2}$ and $f_{3}$ are ambiguous acts.

Let us now come to the DM process. The entity $\Omega_{DM}$ is prepared by the questionnaire in the initial state $p_{v_{0}}$ represented by the unit vector $|v_{0}\rangle =\frac{1}{\sqrt{3}}\left(1,1,1\right)$, which leads to uniform drawing probabilities. However, in the pondering between $f_{1}$ and $f_{2}$, a first context operates on $\Omega_{DM}$ and transforms $p_{v_{0}}$ into a new state $p_{v_{12}}$, depending on individual attitudes towards ambiguity; analogously, in the pondering between $f_{3}$ and $f_{4}$, a second context operates on $\Omega_{DM}$ and transforms $p_{v_{0}}$ into a new state $p_{v_{34}}$, again depending on individual attitudes towards ambiguity. To reproduce Ellsberg preferences, we need to determine two ambiguity averse final states $p_{w_{1}}$ and $p_{w_{2}}$, represented by the unit vectors $|w_{1}\rangle$ and $|w_{2}\rangle$, respectively, such that $W_{w_{1}}\left(f_{1}\right)>W_{w_{1}}\left(f_{2}\right)$ and $W_{w_{2}}\left(f_{4}\right)>W_{w_{2}}\left(f_{3}\right)$. One can show that, for every $\alpha >\frac{1}{\sqrt{3}}$, the unit vectors

(83) $$|w_{1}\rangle =\left(\frac{1}{\sqrt{3}},\alpha ,-\sqrt{\frac{2}{3}-\alpha^{2}}\right)$$

and

(84) $$|w_{2}\rangle =\left(\frac{1}{\sqrt{3}},-\sqrt{\frac{2}{3}-\alpha^{2}},\alpha \right)$$

reproduce Ellsberg preferences [80]. The orthogonality condition $\langle w_{1}|w_{2}\rangle =0$ implies α=±0.7887. We choose α=+0.7887, thus getting

(85) $$\begin{array}{ccc} |w_{1}\rangle & = & (0.5774,0.7887,-0.2113), \end{array}$$

(86) $$\begin{array}{ccc} |w_{2}\rangle & = & (0.5774,-0.2113,0.7887), \end{array}$$

which completes the quantum mathematical modelling of the Ellsberg three-color example.

Let us now represent in Hilbert space the data on the three-color example in Reference [59]. The decision measurement between acts $f_{1}$ and $f_{2}$ is represented by the spectral family {M,1−M}, M=|m〉〈m|, with $∥|m\rangle ∥=1$. From the data in Reference [59], we have $\langle w_{1}|M|w_{1}\rangle =0.82$, and one can show that

(87) $$\begin{array}{c} M=\left(\begin{array}{ccc} 0.333 & 0.396 & 0.256e^{-i105.07^{\circ }}\\ 0.396 & 0.470 & 0.304e^{-i105.07^{\circ }}\\ 0.256e^{i105.07^{\circ }} & 0.304e^{i105.07^{\circ }} & 0.197 \end{array}\right) \end{array}$$

(see Reference [80]). The decision measurement between acts $f_{3}$ and $f_{4}$ is instead represented by the spectral family {N,1−N}, N=|n〉〈n|, with $∥|n\rangle ∥=1$. From the data in Reference [59], we have $\langle w_{2}|N|w_{2}\rangle =0.78$, and one can show that

(88) $$\begin{array}{c} N=\left(\begin{array}{ccc} 0.333 & 0.276e^{-i102.87^{\circ }} & 0.382\\ 0.276e^{i102.87^{\circ }} & 0.229 & 0.317e^{i102.87^{\circ }}\\ 0.382 & 0.317e^{-i102.87^{\circ }} & 0.438 \end{array}\right) \end{array}$$

(see Reference [80]). We have thus completed the construction of a quantum model for the Ellsberg three-color example, which also provides data representation of the DM test in Reference [59]. Ambiguity aversion is one of the conceptual landscapes interacting with the DM entity and changing its state, and genuine quantum effects, namely, contextuality, interference and superposition, occur in the DM process.

The quantum-theoretic framework for human DM under uncertainty can be applied straight to the two-urn example in Section 5. In this case, we have two DM entities, entity $\Omega_{DM}^{I}$, the urn with 100 red and black balls in unknown proportion, and entity $\Omega_{DM}^{II}$, the urn with exactly 50 red balls and 50 black balls. Both $\Omega_{DM}^{I}$ and $\Omega_{DM}^{II}$ are associated with the Hilbert space $C^{2}$. Let {(1,0),(0,1)} be the canonical ON basis of $C^{2}$.

For every i=R,B, the elementary event $E_{i}$ is represented by the one-dimensional orthogonal projection operator $P_{i}=\left|i\rangle \langle i\right|$, where |R〉=(1,0) and |B〉=(0,1). A state $p_{v}$ of both $\Omega_{DM}^{I}$ and $\Omega_{DM}^{II}$ is represented by the unit vector $|v\rangle \in C^{2}$, $∥|v\rangle ∥=1$, that is,

(89) $$|v\rangle =\rho_{R}e^{i\theta_{R}}|R\rangle +\rho_{B}e^{i\theta_{B}}|B\rangle =\left(\rho_{R}e^{i\theta_{R}},\rho_{B}e^{i\theta_{B}}\right)$$

where $\rho_{R},\rho_{B}\geq 0$, $\rho_{R}^{2}+\rho_{B}^{2}=1$, $\theta_{R},\theta_{B}\in R$.

For every i=R,B, the (subjective) probability $\mu_{v}\left(E_{i}\right)$ that the event $E_{i}$ occurs in the state $p_{v}$ is obtained through the Born rule of quantum probability, that is,

(90) $$\mu_{v}\left(E_{i}\right)=\langle v|P_{i}\left|v\rangle =\right|\langle i|v\rangle {|}^{2}=\rho_{i}^{2}.$$

Acts $f_{1}$, $f_{2}$, $f_{3}$ and $f_{4}$ in Table 5 are represented by the hermitian operators

(91) $$\begin{array}{ccc} F_{1} & = & u\left(100\right)P_{R}+u\left(0\right)P_{B}=F_{2}, \end{array}$$

(92) $$\begin{array}{ccc} F_{3} & = & u\left(0\right)P_{R}+u\left(100\right)P_{B}=F_{4}. \end{array}$$

The EU of $f_{1},f_{2},f_{3}$ and $f_{4}$ in a state $p_{v}$ of both $\Omega_{DM}^{I}$ and $\Omega_{DM}^{II}$ is given by

(93) $$\begin{array}{ccc} W_{v}\left(f_{1}\right) & = & \langle v|F_{1}|v\rangle =\rho_{R}^{2}u\left(100\right)+\rho_{B}^{2}u\left(0\right)=\rho_{R}^{2}u\left(100\right)+\left(1-\rho_{R}^{2}\right)u\left(0\right)=W_{v}\left(f_{2}\right), \end{array}$$

(94) $$\begin{array}{ccc} W_{v}\left(f_{3}\right) & = & \langle v|F_{3}|v\rangle =\rho_{R}^{2}u\left(0\right)+\rho_{B}^{2}u\left(100\right)=\rho_{R}^{2}u\left(0\right)+\left(1-\rho_{R}^{2}\right)u\left(100\right)=W_{v}\left(f_{4}\right). \end{array}$$

Let us now come to the DM process. Both $\Omega_{DM}^{I}$ and $\Omega_{DM}^{II}$ are prepared by the questionnaire in the initial state $p_{v_{0}}$ represented by unit vector $|v_{0}\rangle =\frac{1}{\sqrt{2}}|e_{1}\rangle +\frac{1}{\sqrt{2}}|e_{2}\rangle =\frac{1}{\sqrt{2}}\left(1,1\right)$, which leads to uniform drawing probabilities. Then, since $f_{1}$ is ambiguous whereas $f_{2}$ is unambiguous, a pondering between $f_{1}$ and $f_{2}$ will determine a change of $\Omega_{DM}^{I}$ from $p_{v_{0}}$ to a generally different state $p_{w_{1}}$, whereas the same pondering will leave $\Omega_{DM}^{II}$ in the initial state $p_{v_{0}}$. Similarly, since $f_{3}$ is ambiguous whereas $f_{4}$ is unambiguous, a pondering between $f_{3}$ and $f_{4}$ will determine a change of $\Omega_{DM}^{I}$ from $p_{v_{0}}$ to a generally different state $p_{w_{2}}$, whereas the same pondering will leave $\Omega_{DM}^{II}$ in the initial state $p_{v_{0}}$.

One can then show that, for every $0\leq \alpha <\frac{1}{2}$, the unit vectors

(95) $$\begin{array}{ccc} |w_{1}\rangle & = & (\sqrt{\alpha },\sqrt{1-\alpha }), \end{array}$$

(96) $$\begin{array}{ccc} |w_{2}\rangle & = & \left(\sqrt{1-\alpha },-\sqrt{\alpha }\right) \end{array}$$

reproduce Ellsberg preferences $f_{2}≻f_{1}$ and $f_{4}≻f_{3}$ [70]. Indeed, using Equations (93)–(96), we get

(97) $$\begin{array}{ccc} W_{v_{0}}\left(f_{2}\right) & = & \frac{1}{2}\left[u\left(100\right)+u\left(0\right)\right]>\alpha u\left(100\right)+\left(1-\alpha \right)u\left(0\right)=W_{w_{1}}\left(f_{1}\right), \end{array}$$

(98) $$\begin{array}{ccc} W_{v_{0}}\left(f_{4}\right) & = & \frac{1}{2}\left[u\left(100\right)+u\left(0\right)\right]>\left(1-\alpha \right)u\left(0\right)+\alpha u\left(100\right)=W_{w_{2}}\left(f_{3}\right). \end{array}$$

Let us finally represent in Hilbert space the data on the two-urn example in Reference [59]. The decision measurement between acts $f_{1}$ and $f_{2}$ is represented by the spectral family {M,1−M}, where M=|m〉〈m| and $∥|m\rangle ∥=1$. The decision measurement between acts $f_{3}$ and $f_{4}$ is instead represented by the spectral family {N,1−N}, where N=|n〉〈n| and $∥|n\rangle ∥=1$.

From the data in (Aerts, Geriente, Moreira, Sozzo 2018), we have:

(99) $$\begin{array}{ccc} \langle w_{1}|M|w_{1}\rangle & = & 0.82, \end{array}$$

(100) $$\begin{array}{ccc} \langle w_{2}|N|w_{2}\rangle & = & 0.84. \end{array}$$

One can show that the unit vectors

(101) $$\begin{array}{ccc} |w_{1}\rangle & = & (0.38,0.92), \end{array}$$

(102) $$\begin{array}{ccc} |w_{2}\rangle & = & (0.92,-0.38), \end{array}$$

and the one-dimensional orthogonal projection operators

(103) $$\begin{array}{ccc} M & = & \left(\begin{array}{cc} 0.05 & 0.21i\\ -0.21i & 0.95 \end{array}\right), \end{array}$$

(104) $$\begin{array}{ccc} N & = & \left(\begin{array}{cc} 0.93 & -0.13i\\ 0.13i & 0.02 \end{array}\right) \end{array}$$

reproduce empirical data (see References [70,79] for the details). The construction of a quantum model for the Ellsberg two-urn example, which reproduces data in Reference [59], is thus completed. However, the quantum model also works for shifts of ambiguity attitudes, as due to hope and fear effects, in managerial and medical decisions, as proved in Reference [79].

We conclude this section with a remark. According to SEUT, people should take decisions in such a way to maximize EU with respect to a Kolmogorovian probability measure (cfr., Section 5). The analysis provided here shows instead that the paradoxes of SEUT disappear in a theoretical framework in which people actually take decisions in such a way to maximize EU with respect to a non-Kolmogorovian, specifically quantum, probability measure. This suggests to extend EUT in a Hilbert space formalism along the lines suggested in this paper.

## 9. Conclusions

In this paper, we have addressed the question of the foundations of quantum theory as a general theory for human cognition, that is, the problem of how and why we are justified in applying quantum models in cognitive domains independently of their empirical effectiveness. To this end, we have worked out a unitary theoretical framework, inspired by realistic-operational foundations of quantum physics, to represent in Hilbert space the phenomenology of human judgements and DM under uncertainty. In it, a cognitive test is described as a two-step process which involves both a preparation and a measurement on a conceptual entity, while the participant operates in the test as a measurement context for the entity and changes its state. We note the problem of the measurement with respect to the calculation in quantum theory. Consequently, both the problem of quanta observational error of the energy measurement and the inevitable perturbation of the measurement itself arise. On the subject, including Einsteinian *objectivity–and–realism* of a quantum theory are nowadays well identified from a historical–epistemological standpoint. A differ point of view was provided by Dirac. Both scientists essentially concluded that, at that time, the theory was not mature yet to go into a radical solution. For example, why mathematicians and physicists did not perceive that the change of energy could be per quanta? (see, e.g., References [81,82,83,84] and references therein). Whenever the conceptual entity, its states, contexts, properties, and mutual statistical relations are identified in the modelling of a given phenomenon, the quantum-theoretic framework consists in making explicit the usual Hilbert space representation of entities, states, contexts, properties, probabilities and dynamics. We have applied this general theory to long-standing cognitive fallacies, but the theory is in principle applicable to any judgement and decision. The latter remark is important, because the quantum models following from the general framework presented here work, not only in empirical situations where deviations from classicality occur, but also in those situations which admit a classical logical and probabilistic modelling, hence no fallacy occur (see, e.g., classical conjunction and disjunction data in concept combinations [22,35]).

We conclude this section with some remarks that are important, in our opinion, to better grasp the content of this paper.

Firstly, we are aware that a SCoP formalism description contains a degree of idealization and that the large variability in individual differences may play a fundamental role in cognitive processes, which may even mine the Hilbert space representation of some of these processes (see, e.g., Reference [85] for an example of a quantum model that incorporates individual differences). Nevertheless, we also think that a refinement in the description of cognitive processes which takes into account individual differences is not incompatible with a SCoP formalism description where (i) each individual is confronted with a conceptual entity preliminarily prepared by a cognitive test in a defined state, and (ii) the individual acts as a context for the entity, changing its state in a way that is uncontrollable and unpredictable if not at a statistical level. In this regard, it is worth mentioning that we have recently studied processes where effects of memory, evidence accumulation and response dynamics occur in sequential judgements. In these cases, we have proved that, while a general model in Hilbert space cannot be constructed which agrees with empirical data, however, a SCoP formalism description can still be worked out which suggests using a non-Kolmogorovian non-quantum model of probability, called “general tension-reduction (GTR) representation” (see, e.g., Reference [69] and references therein).

Secondly, we do not claim that a “quantum, or quantum-like, theory in Hilbert space” is the only possible theory for human cognition. And, indeed, some authors have proved that suitable classical models where noise is postulated at individual level enable representing a variety of empirical situations where cognitive fallacies occur [86]. On the other side, we also believe that in these cases of empirical equivalence between different scientific paradigms, also epistemological arguments should be taken into account. In this regard, the fact that the present quantum-theoretic framework enables explaining several seemingly different cognitive phenomena in a unitary, cross-disciplinary and coherent structure, using a minimum number of theoretical hypotheses, are serious epistemological arguments in favour of a non-Kolmogorovian quantum, or quantum-like, paradigm.

Thirdly, there is a tendency, mainly in empirically-based disciplines, to be critical with respect to a theory that is able to reproduce all possible situations it applies to. This is because the theory contains additional parameters, which may lead one to think that “any type of data can be modelled by allowing these parameters to get different values”. We agree that, in case we have to do with an “ad hoc model”, that is, a model especially designed for the circumstance of the situation it models, this suspicion is grounded. Adding parameters to such an “ad hoc model”, or stretching the already contained parameters to other values, does not give rise to what we call a theory. On the other hand, a theory needs to be well defined, its rules, the allowed procedures, its theoretical, mathematical, and internal logical structure, “independent” of the structure of the models representing specific situations that can be coped with by the theory. Hence, the theory needs to contain a well defined set of instructions of “how to produce models for specific situations”. In this regard, we distinguish between a model that is derived by a general theory, as the ones presented in this paper, and a model specifically designed to reproduce a number of empirical situations. This is connected with the important issue of the “predictive power” of quantum models. We refer here to the distinction between the scientific explanation—in certain epistemological studies also identified with a prediction—and the description of a scientific account. This distinction is linked with the presence of probabilistic models which can assume an epistemic role both for the explanatory and the descriptive. The former deals with experiments, observables, and so forth, the latter deals with data. Of course, within a scientific process, the activities are connected in a double sense with data. Thus, the explanation–and–description remains an ambiguous object to solve, especially in cognitive and social sciences where, as mentioned above, the model plays a surrogate important role in the final decision (observable–data–information) of the theory. For example, in quantum theory a complex wave function (space and time variables) describes mathematically the state of a (physical complex vector space) system. For, probabilistic models calculate (so they do not measure) the probabilities of a state in a given space and time. This was, of course, not possible in the predictive Newtonian science until 19th century; the same remark applies to Lagrangian formulation [87,88]. Hence, a result is that it is not possible to produce simultaneous measure (and so predictions) of position and momentum, as conjugate variables, to arbitrary precision such as established by Heisenberg’s uncertainty principle. In other words, an electron probabilistically can be located in space but without an exact (prediction of the) position. Therefore, quantum theory, which cannot assign definite values to its variables – by a probabilistic distribution – can scientifically calculate, so describes–and–explains, the values of certain observables. Models derived from a theory will generally need more data from a larger set of tests to become predictive for the outcomes of other not yet performed experiments than this is the case for “ad hoc models”. The reason is that in principle such models have to be able to faithfully represent data of all possible tests that can be performed. In quantum cognition, the relative exiguity of data, in particular, if compared to physics, prevents models from having systematic and substantial predictive power. However, we also believe that the independent applicability of the quantum-theoretic framework presented here constitutes in itself a strong argument to support this line of the quantum cognition research programme. 

## Figures and Tables

**Table 1:** Test by Fisk and Pidgeon on the conjunction fallacy [50]. Values obtained from the quantum model in Section 7.1 are also reported.

Case	μ(A)	μ(B)	μ(AandB)	$\theta_{c}$	|A〉	$e^{-i\theta_{c}}|B\rangle$
LL	0.84	0.62	0.71	94.65	(0.92, 0, 0.4)	(0.27, 0.74, −0.62)
LL	0.59	0.85	0.63	111.28	(0.77, 0, 0.64)	(0.32, 0.86, −0.39)
LU	0.76	0.28	0.37	111.15	(0.87, 0, 0.49)	(0.48, 0.23, −0.85)
LU	0.85	0.31	0.42	119.82	(0.92, 0, 0.39)	(0.35, 0.43, −0.83)
UU	0.33	0.11	0.13	118.19	(0.82, 0, 0.57)	(−0.23, −0.91, 0.33)

**Table 2:** Test by Fisk on the disjunction fallacy [51]. Values obtained from the quantum in Section 7.1 are also reported.

Case	μ(A)	μ(B)	μ(AorB)	$\theta_{d}$	|A〉	$e^{-i\theta_{d}}|B\rangle$
LL	0.82	0.6	0.78	74.88	(0.91, 0, 0.42)	(0.3, 0.72, −0.63)
LL	0.85	0.62	0.72	93.60	(0.92, 0, 0.39)	(0.26, 0.74, −0.62)
LU	0.61	0.2	0.47	79.28	(0.62, 0, 0.78)	(−0.56, −0.7, 0.45)
LU	0.82	0.31	0.62	81.02	(0.91, 0, 0.42)	(0.39, 0.4, −0.83)
UU	0.36	0.11	0.26	82.78	(0.8, 0, 0.6)	(−0.25, −0.91, 0.33)
UU	0.3	0.08	0.23	75.04	(0.84, 0, 0.55)	(−0.19, −0.94, 0.28)

**Table 3:** Average data on the two-stage gamble tests by Tversky and Shafir (TS 1992) [11], Kühberger, Kamunska and Perner (KKP 2001) [52], and Lambdin and Burdsal (LB 2007) [53] on the disjunction effect. Values obtained from the quantum in Section 7.2 are also reported.

Test	μ(A)	μ(B)	μ(AorB)	$\theta_{d}$	|A〉	$e^{-i\theta_{d}}|B\rangle$
TS 1992	0.69	0.58	0.37	137.26	(0.73, 0, 0.68)	(0.61, 0.45, −0.66)
KKP 2001	0.72	0.47	0.48	107.37	(0.85, 0, 0.53)	(0.45, 0.51, −0.73)
LB 2007	0.63	0.45	0.41	106.75	(0.79, 0, 0.61)	(0.57, 0.36, −0.74)

**Table 4:** Payoff table for the Ellsberg three-color example.

	$p(E_{R})=1/3$	$p\left(E_{Y}\right)+p\left(E_{B}\right)=2/3$	
**Act**	**Red**	**Yellow**	**Black**
$f_{1}$	$100	$0	$0
$f_{2}$	$0	$0	$100
$f_{3}$	$100	$100	$0
$f_{4}$	$0	$100	$100

**Table 5:** Payoff matrix for the Ellsberg two-urn example.

	Urn I		Urn II	
**Acts**	$E_{R}$ **: Red Ball**	$E_{B}$ **: Black Ball**	$E_{R}$ **: Red Ball**	$E_{B}$ **: Black Ball**
	$p\left(E_{R}\right)\in \left[0,1\right]$	$p\left(E_{B}\right)=1-p\left(E_{R}\right)$	$p(E_{R})=1/2$	$p(E_{B})=1/2$
$f_{1}$	$100	$0		
$f_{2}$			$100	$0
$f_{3}$	$0	$100		
$f_{4}$			$0	$100
